# Do sequential lineups impair underlying discriminability?

**DOI:** 10.1186/s41235-020-00234-5

**Published:** 2020-08-04

**Authors:** Matthew Kaesler, John C. Dunn, Keith Ransom, Carolyn Semmler

**Affiliations:** 1 University of Adelaide, North Terrace, Adelaide, SA 5005 Australia; 2 University of Western Australia, Crawley, Australia; 3 Edith Cowan University, Joondalup, Australia

**Keywords:** Eyewitness identification, Signal detection model, Lineups, Simultaneous lineup, Sequential lineup

## Abstract

Debate regarding the best way to test and measure eyewitness memory has dominated the eyewitness literature for more than 30 years. We argue that resolution of this debate requires the development and application of appropriate measurement models. In this study we developed models of simultaneous and sequential lineup presentations and used these to compare these procedures in terms of underlying discriminability and response bias, thereby testing a key prediction of diagnostic feature detection theory, that underlying discriminability should be greater for simultaneous than for stopping-rule sequential lineups. We fit the models to the corpus of studies originally described by Palmer and Brewer (2012, *Law and Human Behavior, 36*(3), 247–255), to data from a new experiment and to eight recent studies comparing simultaneous and sequential lineups. We found that although responses tended to be more conservative for sequential lineups there was little or no difference in underlying discriminability between the two procedures. We discuss the implications of these results for the diagnostic feature detection theory and other kinds of sequential lineups used in current jurisdictions.

## Significance statement

Sequential lineups are currently used by police jurisdictions in the USA, Canada and the United Kingdom. Contrary to prior consensus, recent research employing signal detection measures has reported that simultaneous lineups may be superior to sequential lineups. This is consistent with diagnostic feature detection theory (DFDT), which attributes this difference to the greater ability of witnesses presented with a simultaneous lineup to compare different items and to isolate features that are uniquely shared by the perpetrator and the target item. If the sequential lineup is inferior, this has important implications for procedural fairness in those jurisdictions that currently rely on it. In addition, to the degree that this supports theories such as DFDT, these can be used to develop improved lineup procedures that maximize performance. We developed a set of formal models based on signal detection theory and applied them to comparative data drawn from historic and contemporary studies in order to compare underlying memory performance between simultaneous and sequential lineups. Our results revealed little to no simultaneous advantage in underlying discriminability, although the effect may be smaller than our study could detect, and a substantial shift in response bias in that eyewitnesses given sequential lineups require more evidence to identify an item. We show that the reason our results differ from some that have been published previously is due to the way in which eyewitness performance is measured in those studies where they are susceptible to distortion by structural features of the procedures. We also provide supplemental materials for fitting a signal detection model to simultaneous lineup data.

## Overview

A major goal of eyewitness research is to develop procedures that maximize correct identifications and minimize incorrect identifications by eyewitnesses. The sequential lineup has been proposed as one such procedure (Lindsay & Wells, 1985). In contrast to the more traditional simultaneous lineup, in which all items are presented to the eyewitness at the same time, items in the sequential lineup are presented one at a time. Past research had suggested that the sequential lineup is superior to the simultaneous lineup because it leads to a reduced number of incorrect identifications without affecting the number of correct identifications (e.g. Wells, Memon, & Penrod, 2006), suggesting that memory for the perpetrator is expressed more efficiently in the sequential lineup. However, recent studies have drawn the opposite conclusion, finding that simultaneous presentation is superior (e.g., Clark, 2012; Mickes, Flowe, & Wixted, 2012). This raises the question of whether memory for the perpetrator is greater in the sequential lineup compared to the simultaneous lineup or vice versa. In order to answer this question, we argue that it is necessary to apply formal models specific to each procedure in order to measure underlying memory strength and response bias. Our aim in this paper is to develop such models and to apply them to both existing and new data to answer the question of whether memory is the same or different between simultaneous and sequential lineups.

### The sequential lineup

Lineups are typically presented simultaneously, with all lineup items shown at the same time in a single array. A witness may either identify an item as the target (i.e., corresponding to their memory of the perpetrator) or reject the lineup, indicating that no item is a suitable match. In a sequential lineup, as originally proposed by Lindsay and Wells (1985), each lineup item is presented one at a time and, for each item, the witness is asked to judge if it matches their memory of the target by making a “yes/no” judgement. If the witness responds “yes”, the procedure terminates and the remaining lineup items (if any) are not shown. If they respond “no”, they are shown the next lineup item if there is one. The lineup is implicitly rejected if the witness responds “no” to all available lineup members. Variations of this procedure have also been proposed, which do not enforce the immediate stopping rule. These alternatives may permit witnesses to see remaining lineup members after an identification is made (Wilson, Donnelly, Christenfeld, & Wixted, 2019), require witnesses to view all lineup members before making an identification, or allow (or require) witnesses to lap through the procedure a second time (Horry, Brewer, Weber, & Palmer, 2015; Seale-Carlisle, Wetmore, Flowe, & Mickes, 2019).

Lindsay and Wells (1985) originally proposed the sequential lineup based on a theoretical distinction between absolute and relative judgement strategies (Wells, 1984). A relative judgement is said to occur when a witness selects the lineup item most similar to their memory of the target *relative* to the other items. Such a strategy would tend to lead to a high false positive rate because there is a basis for identification even when memory of the perpetrator is poor or the target is not a member of the lineup. An absolute judgement is said to occur when an identification judgement does not depend on the similarity of other lineup items to the witness’ memory of the target. Such a strategy would tend to lead to lower false positive rates because witnesses have a basis to reject the lineup when memory of the target is poor or if the target is not present. Lindsay and Wells (1985) suggested that the sequential lineup would encourage an absolute decision strategy by removing the opportunity to compare lineup items. Consistent with this, Lindsay and Wells (1985) found that sequential presentation led to significantly fewer innocent suspect identifications than simultaneous presentation, accompanied by a relatively small reduction in target identifications. This pattern of results, termed the sequential superiority effect, has been identified in many subsequent studies and in two meta-analyses (Steblay, Dysart, Fulero, & Lindsay, 2001; Steblay, Dysart, & Wells, 2011). Based on this evidence, researchers have successfully advocated a policy shift toward sequential presentation, which has led to its adoption in various forms in 30% of US jurisdictions and in Canada and the United Kingdom (Police Executive Research Forum, 2013; Seale-Carlisle & Mickes, 2016).

### Diagnostic feature detection theory

The interpretation of the sequential superiority effect has recently been challenged by Wixted and Mickes (2014). They have proposed the diagnostic feature detection theory (DFDT) of lineup identification, which predicts a memory advantage for simultaneous lineups compared to sequential lineups. According to this theory, correct identification (and rejection) of a lineup is based on identifying diagnostic features of the different lineup items. A diagnostic feature is one that is uniquely shared by a lineup item and the witness’ memory of the target which, if identified, would support a correct identification. A non-diagnostic feature is one that is shared by all lineup items (e.g. hair colour) which, even if it matches the witness’ memory of the target, cannot support a correct identification. Wixted and Mickes (2014) argued that because a witness is better able to compare the features of different lineup items in a simultaneous lineup, they are better able to identify features that are diagnostic and to discount those that are not.

The distinction between absolute and relative identification strategies proposed by Lindsay and Wells (1985) and DFDT make opposite predictions on the relative merits of simultaneous and sequential lineups - both cannot be correct. This has led to a re-evaluation of the sequential superiority effect and a re-examination of how eyewitness performance is measured. Specifically, researchers have argued that much of the early research on the sequential lineup has obscured potential shortcomings of the sequential procedure by treating the accompanying small reduction in perpetrator identifications as inconsequential (Clark, 2012; Moreland & Clark, 2016). In addition, recent research, employing receiver operating characteristic (ROC) analysis derived from signal detection theory, has found evidence that simultaneous presentation may, in fact, outperform sequential presentation (e.g. Carlson & Carlson, 2014; Dobolyi & Dodson, 2013). We discuss each of these issues in turn.

### Measuring identification performance

In many earlier studies of the sequential superiority effect, eyewitness performance was measured using the diagnosticity ratio statistic, defined as the ratio of the proportion of correct target identifications (TIDs) (the TID rate) to the proportion of incorrect innocent suspect identifications (SIDs) (or the false positive rate). A TID is made when the witness correctly identifies the target in the lineup. An SID is made when the target is not a member of the lineup and the witness incorrectly identifies the innocent suspect. On this measure of performance, an identification made from a lineup procedure that reliably generates a higher diagnosticity ratio is to be preferred to one that does not.

An alternative performance measure is based on signal detection theory (Wixted & Mickes, 2012, 2015a, 2015b) and proposes that performance should be judged in terms of the level of correct identifications that can be obtained for a given level of incorrect suspect identifications. This is termed empirical discriminability and it minimizes the two kinds of identification error discussed previously (Wixted & Mickes, 2018). Empirical discriminability can be measured by constructing an ROC curve. In the context of lineup tasks, this is a plot of TID rates against SID rates at different levels of response bias - the general willingness of a decision-maker to make an identification. In perceptual research, different levels of response bias are achieved by varying payoffs that differentially weight correct and false positive responses, leading decision-makers to be biased towards one kind of response over another. Post-decision confidence estimates are used as a proxy for different levels of response bias in many recognition memory experiments. These may be recorded on a Likert scale or a 0–100% scale with the number of bins set by the researcher.

**Fig. 1:**
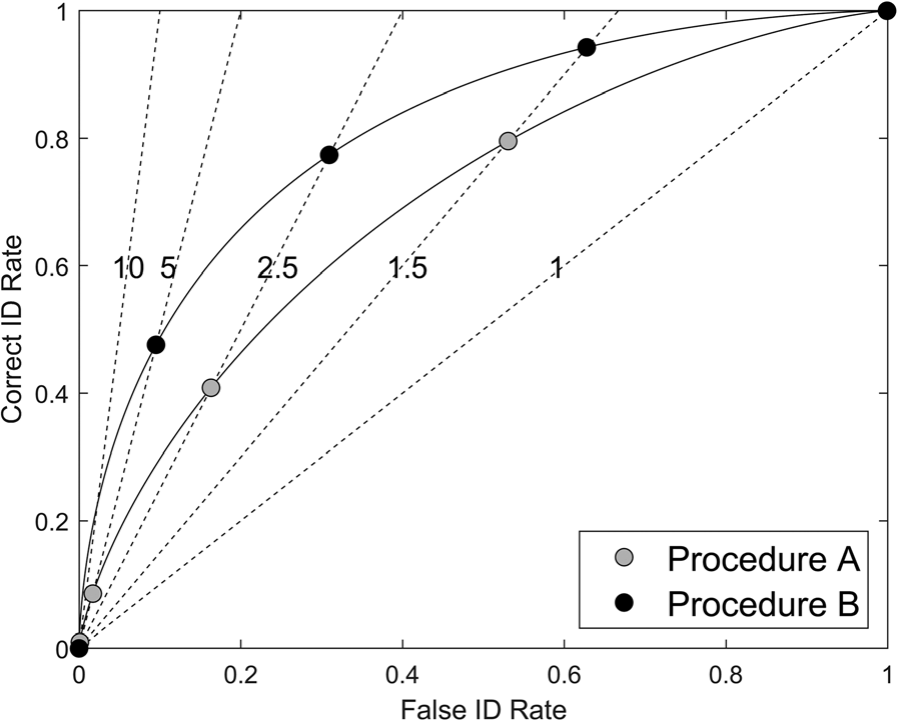
Hypothetical ROC curves for two memory test procedures. Procedure B has higher empirical discriminability (AUC) than Procedure A. The dashed lines represent different diagnosticity ratios taking the values 1, 1.5, 2.5, 5 and 10. Each point on each ROC curve that intersects with a line has the corresponding diagnosticity ratio

Figure 1 displays ROCs for two hypothetical show-up procedures. A show up is a lineup consisting of only one item. These ROC curves have the same form as found in laboratory-based yes-no recognition memory tasks, extending from the extreme lower left to the extreme upper right. The two curves in Fig. 1 differ in empirical discriminability, which is greater for the curve that is closer to the top-left corner. This curve, corresponding to Procedure B in this example, always has a higher correct identification rate for any given incorrect identification rate. If empirical discriminability is zero, the ROC curve falls on the main diagonal indicating chance performance. Following this logic, empirical discriminability can be measured by calculating the area under the ROC curve (AUC). The greater the AUC, the greater the empirical discriminability. The AUC measure is independent of response bias because any combination of correct and incorrect identification rates on the same ROC curve is associated with the same AUC. Accordingly, because Procedure B has greater AUC than Procedure A, it has greater empirical discriminability.

Each point on the ROC curve corresponds to a different response bias and is associated with a given diagnosticity ratio. It is here that the contrast between empirical discriminability and the diagnosticity ratio becomes apparent - the same ratio can be found on different ROC curves corresponding to different levels of discriminability (Gronlund, Wixted, & Mickes, 2014; Rotello, Heit, & Dubé, 2015). This feature is shown in Fig. 1 by the set of dashed lines each of which corresponds to a different diagnosticity ratio (1.0, 1.5, 2.5, 5.0, or 10.0). As can be seen, these lines intersect each of the two ROC curves at different points showing that, all else being equal, the more conservative the response bias (associated with lower false positive rates), the larger the diagnosticity ratio. It is clear from this that the diagnosticity ratio is simply a measure of response bias, independent of empirical discriminability.

### Task dependence of ROC curves

Empirical discriminability provides an objective criterion against which different lineup procedures may be compared. On this view, any procedure that leads to a higher correct identification rate for any given false positive rate is to be preferred (Wixted & Mickes, 2012). However, DFDT is concerned with *underlying* discriminability, i.e. memory strength (Wixted & Mickes, 2018). It proposes that the feature detection mechanism facilitated by simultaneous presentation leads to greater underlying discriminability compared to sequential presentation, and that this explains the superior empirical discriminability of simultaneous presentation observed in some studies using ROC analysis (e.g. Carlson & Carlson, 2014)*.* ROC analysis may be uninformative with respect to underlying discriminability when the procedures being compared have different structural characteristics. In this case, the shapes of the ROC curves and the resulting empirical discriminability associated with each procedure may differ substantially even when underlying discriminability is the same (Rotello & Chen, 2016; Stephens, Dunn, & Hayes, 2019).

**Fig. 2:**
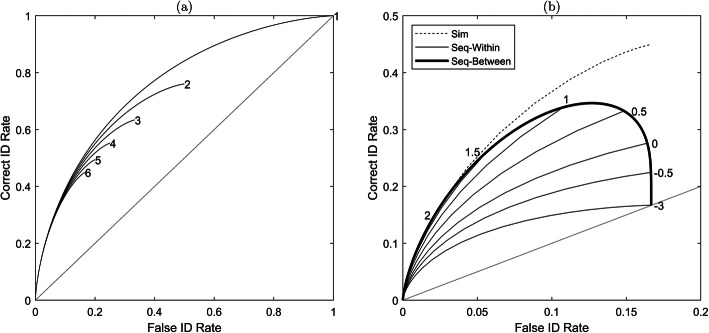
The effect of task characteristics on the shapes of ROC curves. **a** The set of ROC curves for simultaneous lineups of sizes 1 (show up) to 6 as indicated on the figure. Each curve is associated with the same underlying *d*_*t*_ = 1. **b** The set of ROC curves for simultaneous and sequential lineups of size 6 and *d*_*t*_ = 1. The dashed line is the ROC curve for a simultaneous lineup, identical to curve 6 in panel **a**. The thin solid lines are the ROC curves for a sequential lineup associated with different minimum criteria to choose, ranging from conservative (2) to lenient (−3). The thick solid line is the ROC curve for a sequential lineup in which response bias is manipulated between participants and ranges from most conservative to most lenient

A dissociation between empirical and underlying discriminability due to structural features of a task is illustrated in Fig. 2a. This shows a family of hypothetical ROC curves derived from lineups of different sizes. These curves were generated using the simultaneous lineup model signal detection theory (SDT)-MAX, which we define later (the relevant formulas are given in Additional file 1). This model is based on a signal detection framework in which there is a normal distribution of familiarity values for the target item and another normal distribution for foil items, including the innocent suspect. For each lineup size, although underlying discriminability (i.e. the difference between the familiarity distributions of the target and foils) is the same, the shape and termination point of each ROC curve is different. Each curve terminates at a different point because, under the most lenient response bias (i.e. always select a lineup member) there is a 1/*n* chance of choosing the innocent suspect, where *n* is the lineup size. Thus, because *n* differs between the curves, each must terminate at a different point corresponding to a false positive rate of 1/*n*.

Because the ROC curves in Fig. 2a were all generated from the same underlying signal detection model, the differences are due to a structural characteristic of the lineup task - specifically the lineup size. This means that differences in empirical discriminability between these tasks do not indicate differences in underlying discriminability (which is the same for each curve).

From the foregoing, it should come as no surprise that structural characteristics of the sequential lineup also change the shape of the ROC curve. In this case, it is not the size of the lineup that is critical, but the minimum level of evidence required to make an identification. Figure 2b shows a set of ROC curves for a sequential lineup of size 6, each constructed with a different minimum level of evidence. The ROC curves shown by thin solid lines in Fig. 2b illustrate different choices for the minimum level of evidence expressed in terms of a decision criterion on the familiarity axis. The value of this criterion is indicated at the end of each corresponding ROC curve. A large value indicates a conservative response bias for which a relatively high level of familiarity is required for a lineup item to trigger identification. A small value indicates a lenient response bias for which a relatively low level of familiarity is sufficient to trigger identification. Each of these ROC curves terminates at a different point. In the limit, when the minimum evidence is very low, the ROC curve terminates on the main diagonal (indicated by the dotted line in Fig. 2b). The ROC curve shown by the thick solid line corresponds to the situation in which each witness has a different level of minimum evidence. It encloses the set of confidence-based ROC curves and is clearly non-monotonic. Rotello and Chen (2016) observed a similar shaped curve in their simulations of the sequential lineup, as did Wilson et al. (2019) in empirical sequential lineup data.

Figure 2b also shows the ROC curve generated from a simultaneous lineup of size 6 as shown in Fig. 2a (by the curve labelled 6). Altogether, these curves show that even when underlying discriminability is held constant, the shapes of ROCs and the corresponding empirical discriminability values differ to a considerable degree. It is therefore important to distinguish two research questions. One question is about empirical discriminability - for any given false identification rate, which procedure leads to higher correct identification rates? The ROC curves shown in Fig. 2a and b suggest that simultaneous lineups are preferred to sequential lineups and, within the class of simultaneous lineups, smaller lineup sizes are preferred to larger lineup sizes. Empirical research also supports this conclusion, at least with respect to simultaneous, as compared to sequential lineups (Carlson & Carlson, 2014; Dobolyi & Dodson, 2013; Experiment 1a Mickes et al., 2012; Neuschatz et al., 2016), although this has not always been found (Flowe, Smith, Karoglu, Onwuegbusi, & Rai, 2016; Gronlund et al., 2012; Experiment 1b and 2 Mickes et al., 2012; Sučić, Tokić, & Ivešić, 2015).

The second question bears on DFDT and concerns underlying discriminability - which eyewitness test procedure reveals higher levels of memory strength? ROC curves and the AUC cannot be used to answer this question. As shown above, they may not reflect underlying discriminability across different lineup procedures. In order to measure underlying discriminability, it is necessary to use a formal model to measure the parameter of interest. In this section we outline two models of the simultaneous lineup task based on signal detection theory (SDT-MAX and SDT-INT) and develop a comparable model of the simple stopping rule version of the sequential lineup task (SDT-SEQ). We then apply these models to extant and new data to estimate memory strength across the two procedures.

### Unequal variance signal detection model

The starting point for all the lineup models we consider is the unequal variance signal detection (UVSD) model. The UVSD model accounts well for data in laboratory-based recognition memory tests (Jang, Wixted, & Huber, 2009; Mickes, Wixted, & Wais, 2007) and can be extended to account for lineup tasks. In a typical eyewitness experiment, a participant views a simulated crime conducted by a perpetrator and is subsequently shown an *n*-item lineup. In a target present (TP) lineup, one item is the *target* (a picture of the perpetrator) and the remaining items are foils or fillers (pictures of other people). In a target absent (TA) lineup, one item may be designated as the innocent *suspect* with the remaining items being foils. The participant is required to judge whether the lineup contains the target and, if they believe it does, to identify the corresponding item. We assume that each lineup item is associated with a familiarity value that reflects its similarity to the participant’s memory of the perpetrator. Each familiarity value is considered a random draw from one of several distributions - a target distribution if the item is a target, an innocent suspect distribution if it is an innocent suspect^1^, and a foil distribution if it is a foil. In order for the models to be testable we assume that each distribution is Gaussian. Consistent with most signal detection models, the foil distribution is assigned a mean of zero and a standard deviation of one. The target distribution has mean *d*_*t*_ and standard deviation *s*_*t*_, both of which can be estimated from the data. Because *s*_*t*_ may not equal one the model is called the *unequal variance* signal detection model. In addition, because the innocent suspect may be distinct from the remaining foils, the suspect distribution has mean *d*_*s*_ and standard deviation *s*_*s*_.

A lineup can be considered as a combination of a detection question, “Is the target present?”, and an identification, “If so, which item is the target?” (Duncan, 2006). While the answer to the identification question is relatively straightforward - always choose the lineup member associated with the greatest familiarity - the answer to the detection question is less clear-cut. This leads to different models based on different decision rules. Although there is a wide range of possible decision rules, we consider two in particular, which we call SDT-MAX and SDT-INT. In the SDT-MAX model, the decision rule is to compare the familiarity value of the most familiar lineup item (the maximum) to a response criterion. In the SDT-INT model, the decision rule is to compare the *sum* of the familiarity values of the lineup items to a response criterion. For both of these models, if the relevant value exceeds the criterion, the most familiar item is identified as the target. We also developed a model of the sequential lineup. In this case, because the witness does not see all the lineup items until the end, and may not see all items if they choose before reaching the end, it not possible before that point to identify either the maximum or the sum, or any other function of the familiarity values of the entire lineup. For this reason, we developed a model of the sequential lineup, here called SDT-SEQ.

### SDT-MAX

SDT-MAX, also known as the independent observations model (Duncan, 2006; Wixted, Vul, Mickes, & Wilson, 2018), is perhaps the simplest model of the simultaneous lineup. In this model, identification decisions are made with respect to a set of *k* decision criteria, *C* = {*c*_1_, …, *c*_*k*_} such that *c*_1_ < *c*_2_ < … < *c*_*k*_, that define a set of *k* + 1 confidence levels. Let *X* = {*x*_1_, …, *x*_*n*_} be the set of familiarity values associated with each of *n* lineup items. Let *x*_*m*_ = max(*X*) be the maximum familiarity value associated with item *m*. The decision rule is this: if *x*_*m*_ < *c*_*1*_ then reject the lineup, otherwise choose lineup item *m* with confidence level *l* where *c*_*l*_ is the largest element of the set, {*c*_*i*_ ∈ *C* : *x*_*m*_ ≥ *c*_*i*_}.

As detailed in Additional file 1, we derive general formulas for the probability of a correct identification and the probability of a false identification under the SDT-MAX model. We summarize these below under the assumption that all the underlying distributions are Gaussian. Let *ϕ(x;μ,σ)* be the normal probability density function and let Φ(x;μ,σ) be the normal cumulative distribution function evaluated at *x* ∈ ℝ. Recall that the foil distribution takes the form of the standard normal distribution with *µ* = 0 and *σ* = 1. In this case, we write *ϕ*(*x*;0,1) = *ϕ*(*x*) and ϕ(*x*;0,1) = ϕ(*x*). Let *c* ∈ *C* be a decision criterion and let *P*_*TID*_(*c*) be the probability of a correct target identification with confidence greater than or equal to *c*. Then

$$ {P}_{TID}(c)={\int}_c^{\infty}\phi \left(x;{d}_t,{s}_t\right)\kern0.1em \Phi {(x)}^{n-1} dx. $$

Similarly, let *P*_*SID*_(*c*) be the probability of an incorrect suspect identification with confidence greater than or equal to *c*. Then, if there is a designated innocent suspect,

$$ {P}_{SID}(c)={\int}_c^{\infty}\phi \left(x;{d}_s,{s}_s\right)\kern0.1em \Phi {(x)}^{n-1} dx, $$

otherwise,

$$ {P}_{SID}(c)=\frac{1}{n}\left(1-\Phi {(c)}^n\right). $$

### SDT-INT

Let $\mathrm{sum}(X)$ be the sum of familiarity values of all the lineup items. The decision rule is this: If sum(*X*) < *c*_1_ then reject the lineup, otherwise choose lineup member *m* with confidence level *l* where *c*^*l*^ is the largest element of the set, {*c *∈ *C* : sum(*X*) ≥ *c*} 

The equations for the probability of a correct identification and probability of a false identification under the SDT-INT model are summarized below (see Additional file 1 for details).

$$ {\displaystyle \begin{array}{rl}{P}_{TID}(c)& =\Pr \left(\mathrm{sum}(X)\ge c\mid m=t\right)\cdot \Pr \left(m=t\right)\\ {}& \approx {\int}_{-\infty}^{\infty}\left(1-\Phi \left(c-x;\left(n-1\right){\mu}_x,\sqrt{\left(n-1\right)}{\sigma}_x\right)\right)\phi \left(x;{d}_t,{s}_t\right)\Phi {(x)}^{n-1} dx\end{array}} $$

where *t* is the position of the target item and *μ*_*x*_ and *σ*_*x*_ are the mean and standard deviation, respectively, of the standard normal distribution truncated at the upper limit of *x*. The equation is not exact because it assumes that the sum of truncated distributions is approximately normal (by the Central Limit Theorem). Similarly, if there is a designated innocent suspect, then

$$ {P}_{SID}(c)\approx {\int}_{-\infty}^{\infty}\left(1-\Phi \left(c-x;\left(n-1\right){\mu}_x,\sqrt{\left(n-1\right)}{\sigma}_x\right)\right)\phi \left(x;{d}_s,{s}_s\right)\Phi {(x)}^{n-1} dx, $$

otherwise,

$$ {P}_{SID}(c)=\frac{1}{n}\left(1-\Phi \left(c;0,\sqrt{n}\right)\right). $$

### SDT-SEQ

Our model for sequential presentation is also based on the UVSD framework and incorporates the “first-above-criterion” decision rule where presentation of the lineup items is terminated as soon as an identification is made. As detailed in Additional file 1, we derive the following equations for the probability of a correct identification and probability of a false identification under the SDT-SEQ model. Let *p*_*i*_ be the probability that the item in lineup position *i* is a target. Then

$$ {P}_{TID}(c)=\left(1-\Phi \left(c;{d}_t,{s}_t\right)\right)\sum \limits_{i=1}^n{p}_i\Phi {\left({c}_1\right)}^{i-1}. $$

If there is a designated innocent suspect, let *q*^*i*^ be the probability that the lineup item at position *i* is the suspect. Then,

$$ {P}_{SID}(c)=\left(1-\Phi \left(c;{d}_s,{s}_s\right)\right)\sum \limits_{i=1}^n{q}_i\Phi {\left({c}_1\right)}^{i-1}. $$

otherwise,

$$ {P}_{SID}(c)=\frac{1}{n}\left(1-\Phi (c)\right)\sum \limits_{i=1}^n\Phi {\left({c}_1\right)}^{i-1}. $$

### Palmer and Brewer (2012) database

Palmer and Brewer (2012) conducted an extensive analysis of previously published studies that compared simultaneous and stopping-rule sequential lineups under the same conditions. They fit a signal detection model equivalent to the SDT-INT model described previously, to data from 22 previous studies. Their aim was to determine if either underlying discriminability and/or response bias differs between sequential and simultaneous lineups. Their analysis revealed that, across the datasets, the two presentation methods did not differ in terms of underlying discriminability but that the sequential procedure was associated with more conservative responding.

While the finding of equal underlying discriminability is not consistent with DFDT, the difference in response criteria was consistent with the view that a sequential lineup produces a higher diagnosticity ratio. It is now widely accepted that sequential presentation leads to more conservative responding than simultaneous presentation (Clark, 2012; Clark, Moreland, & Gronlund, 2014; Wells, 2014; Wixted & Mickes, 2014). The apparent success of the modelling approach employed by Palmer and Brewer (2012) has also led researchers to use SDT-INT to examine other aspects of the sequential lineup (Carlson, Carlson, Weatherford, Tucker, & Bednarz, 2016; Horry et al., 2015; Horry, Palmer, & Brewer, 2012).

However, there are aspects of the Palmer and Brewer (2012) approach that challenge the validity of their conclusions. First, and most critically, the SDT-INT model was fit to data from both simultaneous and sequential lineups. No attempt was made to model the unique task demands of sequential presentation. It is therefore unknown whether the same results would be found if a more appropriate model were used, such as SDT-SEQ as described previously. Second, the SDT-INT model does not exhaust the set of decision rules for simultaneous lineups (Wixted et al., 2018). A different decision rule, such as SDT-MAX, may lead to different results. Third, Palmer and Brewer (2012) fit the SDT-INT model using an inefficient and potentially inaccurate manual grid search of parameter space. Finally, because confidence judgements were not available, it was only possible to fit an equal variance signal detection model in which *s*_*t*_ = *s*_*s*_ = 1. If this is not an appropriate model of their data, the results may be distorted.

### Summary and aims

The aim of the present paper was to compare simultaneous and sequential lineups in order to test the central prediction of DFDT that simultaneous presentation is associated with greater underlying discriminability than sequential presentation. To do this, we first re-analysed the corpus of simultaneous and sequential data from Palmer and Brewer (2012), addressing the previously described problems in their analysis. Principally, we fit a model of the sequential lineup, SDT-SEQ, specifically developed for this task, and two models of the simultaneous lineup - the SDT-INT model as used by Palmer and Brewer (2012) and the alternative SDT-MAX model. Third, we fit each model using an efficient optimisation procedure that leads to more accurate solutions. Second, we conducted a new experiment from which we obtained confidence judgements enabling us to fit models based on the assumption of unequal variances.

### Predictions

Predictions were preregistered on the Open Science Framework, available at https://osf.io/xwp9d/. DFDT predicts that simultaneous presentation should lead to greater underlying discriminability than sequential presentation. Specifically, this means that the estimate of *d*_*t*_ (or the difference *d*_*t*_ – *d*_*s*_ if there is a designated suspect) should be greater for simultaneous lineups. Based on the conclusions reached by Palmer and Brewer (2012), sequential presentation is predicted to lead to more conservative responding than simultaneous presentation. This means that the estimate of *c*_1_ (and possibly other criteria) should be greater for sequential lineups.

### Model cross fit

We have described three models that we propose to fit to data. This is motivated in part by the idea that there are differences between the models that determine how well they fit different kinds of data. This means that if data are simulated from a model, while this model should fit the data well, other models should fit relatively poorly. In order to investigate this question, we conducted a cross fitting and parameter recovery analysis. First, we randomly generated 100 sets of parameter values for a 6-item lineup and then used each of these to generate 100 simulated datasets from each model. To avoid issues with low cell counts, we set the number of TP and TA lineups to 10,000, giving 20,000 simulated observations for each dataset. We then fit each model to its own sets of data and to those generated by the other models, recording the *χ*^2^ value, *p* value and parameter estimates from each fit. Further detail on the simulation process and expanded results are available in Additional file 2.

**Fig. 3:**
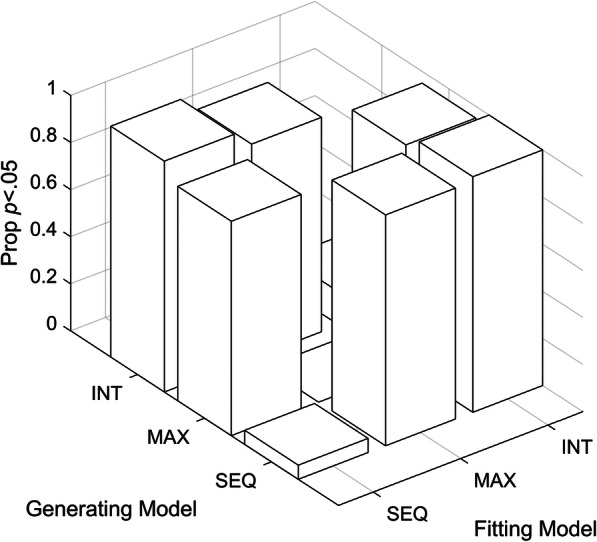
Proportion of *p* < .05 for models cross fit to simulated data. Each model was fit to its own 100 simulated datasets and cross fit to the 100 datasets generated by the other models. The bars show the proportion of datasets for which the models could be rejected at α = .05

Figure 3 shows the proportion of datasets where the model could be rejected at *p* < .05. It shows that when a model is fit to data generated by any other model, it is highly likely to be rejected. In other words, the models are, in principle, distinct - given sufficient statistical power, if the data are consistent with one model then they should be poorly fit by any of the remaining models.

### Parameter recovery

We measured parameter recovery by examining the correlation between generating and recovered parameter values for each model fit. Scatterplots and tables of correlations are available in Additional file 2. We were interested in two aspects of this analysis. First, it is desirable for the correlation value to be close to 1 when the models are fit to their own data. Second, it is also important to understand how well the models recover the correct parameter values when fit to data they did not generate as, in some cases, they may fit well but recover incorrect parameter estimates.

When fit to their own data, the models generally recover their own parameters well, with *r* > = .90 for generating versus recovered parameter values. SDT-MAX recovers the generating parameters perfectly when fit to its own data, but both SDT-SEQ and, to a lesser extent, SDT-INT, recover a small number of outliers, affecting the correlation coefficients. These are most likely due to the presence of local minima, which can be avoided by starting parameter search from different initial values. It is evident from the scatterplots in Additional file 2 that recovery is close to perfect once these outliers are excluded.

When SDT-MAX and SDT-INT are fit to data generated by SDT-SEQ, recovery of *d*_*t*_ is poor. This suggests that if SDT-SEQ is a good representation of the sequential lineup task, then fitting SDT-MAX or SDT-INT to sequential lineup data may lead to inaccurate estimates of *d*_*t*_. Recovery of *s*_*t*_ was poor for all models when fit to data they did not generate, while recovery of the decision criteria (*c*_1_, …, *c*_5_) was generally good for all fits, with *r > =* .80.

## Re-analysis of the Palmer and Brewer (2012) dataset

Palmer and Brewer (2012) selected a corpus of 22 studies (total *N* = 3871, simultaneous *n* = 1952, sequential *n* = 1919) that compared simultaneous and stopping-rule sequential presentation procedures using the “full diagnostic design” inclusion criteria described in Steblay et al. (2011). That is, each study manipulated both presentation format (simultaneous versus sequential) and target presence (present versus absent), reported above-chance identification performance, defined as *P*_*TID*_ − *P*_*SID*_ > 0.1, in at least one of the four experimental conditions, and included only adult participants.

The simultaneous lineup data from each study were fit by both SDT-INT (as undertaken by Palmer and Brewer) and SDT-MAX. The corresponding sequential lineup data were fit by SDT-INT (as undertaken by Palmer and Brewer) and SDT-SEQ. Each model was fit using the Matlab® fmincon function. Because each study required participants to make a single choose-no choose decision, there are not enough degrees of freedom to fit all of the model parameters, specifically *c*, *d*_*t*_, *d*_*s*_, *s*_*t*_, and *s*_*s*_, without the model becoming saturated (i.e., having no remaining degrees of freedom). Accordingly, we specified that *s*_*t*_ = *s*_*s*_ = 1, as was also assumed by Palmer and Brewer.

Some studies designated an innocent suspect while others did not. When a suspect had been designated, we estimated *d*_*s*_, the mean of the suspect distribution, otherwise we stipulated that *d*_*s*_ = 0, the same as the mean of the foil distribution. In addition, studies differed in the probability of a target (and suspect if relevant) appearing at different sequential lineup positions. When specified, this information was used in fitting the SDT-SEQ model (see Additional file 1 for details), otherwise it was assumed that the target/suspect had the same probability of appearing at each lineup position.

### Results and discussion

#### Model fit performance

Table 1 presents the *χ*^2^ goodness-of-fit values for each dataset and each fitted model. Each *χ*^2^ test has one degree of freedom and we set α = .01 to control the type I error rate across the large number of tests conducted. We fit the SDT-MAX and SDT-INT models to the simultaneous lineup data and SDT-SEQ and SDT-INT to sequential lineup data. SDT-MAX fit 20 of 22 simultaneous datasets, as indicated by non-significant *χ*^2^ values. The model did not fit data from two studies - Carlson et al. (2008) experiment (Exp) 2 and Greathouse and Kovera (2009). SDT-INT performed similarly, also failing to fit the two studies above, in addition to Lindsay and Wells (1985). For the sequential lineups, SDT-SEQ fit 19 of 22 data sets, failing to fit data from Kneller et al. (2001), Lindsay and Wells (1985) and Pozzulo and Marciniak (2006). SDT-INT failed to fit the three datasets above, in addition to experiment one and two from Carlson et al. (2008). In all, SDT-MAX and SDT-SEQ performed better than SDT-INT when fit to data from simultaneous and sequential lineups respectively. Similar results with respect to simultaneous lineup data were found by Wixted et al. (2018), who examined the performance of SDT-MAX and SDT-INT by fitting these models to a number of previous lineup datasets.

**Table 1:** χ2 goodness-of-fit values for each dataset, presentation format and model

Dataset	Simultaneous lineup		Sequential lineup	
	SDT-MAX	SDT-INT	SDT-SEQ	SDT-INT
Carlson, Gronlund, and Clark (2008, Exp 1)	.01	2.29	2.01	11.48*
Carlson et al. (2008, Exp 2)	20.81*	36.53*	.23	30.04*
Clark & Davey (2005, Exp 1)	.39	.08	.06	.05
Clark & Davey (2005, Exp 2)	.30	.03	1.14	.51
Greathouse and Kovera (2009)	9.23*	10.28*	2.91	.01
Kneller, Memon, and Stevenage (2001)	2.68	3.20	10.91*	13.17*
Levi (2006)	.08	.72	.17	.10
Lindsay, Lea, & Fulford (1991)	1.24	1.39	.17	4.99
Lindsay and Wells (1985)	5.99	11.86*	6.74*	22.25*
MacLin & Phelan (2007)	.37	.21	.02	.00
MacLin et al. (2005, Exp 1)	.25	.22	1.41	1.39
MacLin et al. (2005, Exp 2)	.61	.46	.00	.03
Melara et al. (1989)	1.14	1.18	.07	.01
Memon & Gabbert (2003)	.31	.48	.05	.34
Parker & Ryan (1993)	1.38	4.33	.00	.27
Pozzulo et al. (2008)	.03	.00	.00	.06
Pozzulo and Marciniak (2006)	.09	.03	12.18*	13.75*
Rose et al. (2005)	.49	1.62	.01	0.10
Sporer (1993)	.66	.63	.63	.44
Steblay et al. (2011)	.72	1.24	.00	.07
Wells & Pozzulo (2006)	.47	.24	.59	.73
Wilcock et al. (2005)	5.34	5.56	.02	.17

*Non-fitting datasets: asterisks indicate a significant difference from zero, α = 0.01 (critical value = 6.63)

We examined the datasets that were not fit by one or more models. Our first observation was that each of these contained a limited number of observations, although this was also true for other datasets that were fit well. Second, in the case of Carlson et al. (2008, Exp 2), Greathouse and Kovera (2009) and Palmer and Brewer (2012), Pozzulo and Marciniak (2006) had collapsed the relevant data across different experimental conditions. In addition to presentation format, Carlson et al. (2008, Exp 2) manipulated lineup fairness, Greathouse and Kovera (2009) manipulated administrator bias and lineup fairness, and Pozzulo and Marciniak (2006) manipulated appearance change from encoding to test. Given that these manipulations may have affected the underlying signal detection parameters and that collapsing across these conditions may have caused the models to perform poorly, we disaggregated each dataset in to its original experimental conditions and re-fit the models to these datasets. The resulting *χ*^2^ values are shown in Table 2, revealing improved model fits in 10 of 18 experimental conditions.

**Table 2:** Chi-square fit values for previously non-fitting datasets, disaggregated in to original experimental conditions

Dataset	Simultaneous lineup		Sequential lineup	
	SDT-MAX	SDT-INT	SDT-SEQ	SDT-INT
Carlson et al. (2008, Exp 2) – biased	19.68*	19.66*	1.94	22.76*
Carlson et al. (2008, Exp 2) – intermediate	.81	3.02	.42	10.23*
Carlson et al. (2008, Exp 2) – fair	10.00*	16.88*	.85	2.61
Greathouse and Kovera (2009) – biased, single-blind	.15	.38	.78	.15
Greathouse and Kovera (2009) – biased, double-blind	.57	.38	4.08	2.17
Greathouse and Kovera (2009) – fair, single-blind	5.44	6.29	4.06	.44
Greathouse and Kovera (2009) – fair, double-blind	3.83	4.09	.25	.15
Pozzulo and Marciniak (2006) – no appearance change	.89	.09	1.82	3.81
Pozzulo and Marciniak (2006) –appearance changed	.21	.06	12.60*	11.58*

*Non-fitting datasets: asterisks indicate a significant difference from zero, α = 0.01 (critical value = 6.63)

#### Parameter estimates

In order to compare our results with Palmer and Brewer (2012), we report parameter values recovered from fitting the models to the same 22-dataset corpus, rather than disaggregating each study in to its original experimental conditions. A full table of parameter estimates is available in Table S1, Additional file 3. Table 3 shows the mean estimates of the model parameters and their standard deviations for each presentation format, weighted by sample size. The parameters are underlying discriminability, decision criterion, *c,* and a derived decision parameter *C*, which Palmer and Brewer (2012) used in their original analysis. *C* is defined as, *C* = *c* − *d*_*t*_/2 , with zero indicating an “unbiased” criterion set at the midpoint between the target and foil distributions. Negative values indicate a lenient response criterion while positive values indicate a conservative criterion. This metric is only relevant in the equal variance case, as a change in target distribution variance will shift the point at which choosing would be truly unbiased. Our hypothesis tests are based on the estimated parameters from fitting SDT-MAX fit to the simultaneous data and SDT-SEQ fit to the sequential data. Mean weighted parameter values from fitting SDT-INT to both data types and as calculated from the original Palmer and Brewer (2012) fits are presented for comparison.

**Table 3:** Mean parameter values weighted by sample size calculated from the estimates reported in Palmer and Brewer (2012) and our reanalysis

Format	Source	Parameter					
		*Discriminability*		*c*		*C*	
		*μ*_*w*_	*σ*_*w*_	*μ*_*w*_	*σ*_*w*_	*μ*_*w*_	*σ*_*w*_
Simultaneous	Palmer and Brewer (2012)	1.64	.50	−.07	.37	−.89	.33
	SDT-MAX	.91	.72	1.24	.24	.58	.25
	SDT-INT	.94	1.02	−.17	.82	−1.01	.72
Sequential	Palmer and Brewer (2012)	1.75	.62	.48	.59	−.38	.49
	SDT-SEQ	.99	.58	1.61	.37	.92	.39
	SDT-INT	.93	.93	1.07	1.37	0.18	1.25

##### Underlying discriminability

**Fig. 4:**
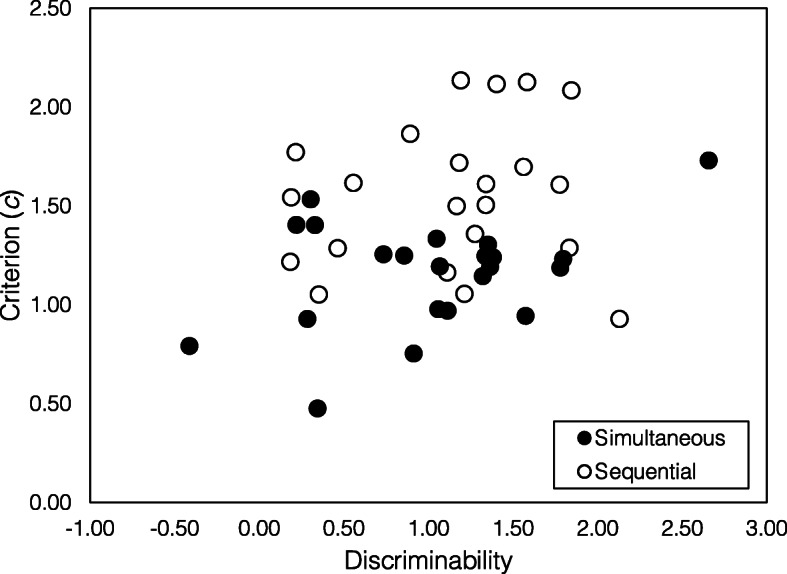
Criterion (*c*) vs discriminability for each dataset in the Palmer and Brewer (2012) corpus. Simultaneous and sequential underlying discriminability and *c* as estimated by SDT-MAX and SDT-SEQ, respectively

Figure 4 shows underlying discriminability plotted against criterion *c* estimated by SDT-MAX and SDT-SEQ fit to simultaneous and sequential lineups, respectively. For studies that specified a designated innocent suspect, underlying discriminability was calculated as *d*_*t*_*– d*_*s*_. Visual examination of Fig. 4 reveals no particular relationship between underlying discriminability and presentation format. Mean weighted underlying discriminability shown in Table 3 does not differ between simultaneous and sequential presentation, as indicated by a Welch two-sample weighted *t* test, *t* (40.33) = .40, *p =*.69. We re-ran the analysis, excluding datasets that the models failed to fit, but this did not change the result. This result is consistent with the conclusion reached by Palmer and Brewer (2012) and fails to support our hypothesis that underlying discriminability is greater for simultaneous presentation.

Table 3 shows that the mean-weighted estimates of underlying discriminability recovered by SDT-INT for each presentation format are similar to those recovered by SDT-MAX and SDT-SEQ when fit to their respective data types. The Welch two-sample weighted *t* test indicated that there is no significant difference for simultaneous, *t* (37.53) = .08, *p =* .94, or sequential presentation, *t* (35.22) = -.26, *p =* .79.

Our estimates of mean weighted underlying discriminability shown in Table 3 are lower than those calculated from the original Palmer and Brewer (2012) analyses and those reported in our preliminary analysis of this corpus (Kaesler, Semmler, & Dunn, 2017). This is because we estimated *d*_*s*_ for studies that employed a designated innocent suspect selected to resemble the perpetrator more closely than foils, where previous analyses assumed that the innocent suspect and the foils were drawn from the same distribution with a mean of zero and standard deviation of one. In the case where *d*_*s*_ is greater than zero, DFDT does not predict a strong simultaneous advantage, because the features uniquely shared by the innocent suspect and the perpetrator will cause the innocent suspect to be identified at a higher rate in the simultaneous procedure compared to the sequential procedure. For this reason, we examined whether there was a simultaneous advantage in the subset of studies that did not use an innocent suspect. We found that the mean weighted difference in underlying discriminability between simultaneous and sequential presentation as estimated by SDT-MAX and SDT-SEQ respectively was less for the 8 studies that used an innocent suspect (*M* = −.23) compared to the 14 that did not (*M* = .09). However, the Welch two-sample weighted *t* test indicated that the difference between these means is not significant, *t* (9.78) = − 1.61, *p =* .14.

##### Response bias

Visual examination of Fig. 4 shows an apparent difference between sequential and simultaneous datasets for values of the decision criterion, *c*. Analysis of mean weighted *c* values show that these are greater (indicating more conservative responding) for sequential than for simultaneous lineups, Welch two-sample weighted *t* test, *t* (35.83) = − 3.88, *p* < .01. Once again, excluding the datasets that the models failed to fit did not change the result.

### Summary

The re-analysis of the Palmer and Brewer (2012) corpus of data reaffirmed their original finding of no significant difference in underlying discriminability between sequential and simultaneous presentation. SDT-MAX and SDT-SEQ performed similarly and recovered similar parameter estimates to SDT-INT when fit to their respective data types. This is in contrast to simulations we conducted that showed that the models behave differently over the entire parameter space. Both of these results may be attributable to low statistical power since each study on average had fewer than 100 participants. It is possible that, because of the relatively small number of participants in each study, each individual analysis lacked the statistical power to detect both differences in the fits of models and differences in underlying discriminability between simultaneous and sequential lineups.

In addition to a lack of statistical power, two other methodological issues limit the utility of the corpus for investigating differences in underlying discriminability. First, a designated innocent suspect was selected to resemble the perpetrator in some studies, which may attenuate any simultaneous advantage in underlying discriminability. Second, the target was fixed to appear in certain positions in many of the sequential lineup studies. While our modelling approach accounted for fixed target positions, there is some evidence to suggest that underlying discriminability may increase with target position (Wilson et al., 2019). As a result, those studies in which the target was fixed to appear late in the lineup may have overestimated underlying discriminability compared to studies in which the target position was randomised. In addition, because each study either recorded or reported only a binary (yes/no) decision, it was necessary to assume an underlying equal variance signal detection model. Although the resulting model fits were good, it is possible that the parameter estimates may have been systematically affected. For these reasons, we conducted a new experiment that sought to address each of these limitations.

## Experiment 1

The aim of experiment 1 is to compare a simultaneous lineup and a stopping-rule sequential lineup, extending the studies examined by Palmer and Brewer (2012), by increasing statistical power using a large sample size, collecting confidence judgements, and avoiding using a designated innocent suspect.

### Design

We employed a 2 × 2 between-participants factorial design, manipulating presentation format (simultaneous versus sequential) and target presence (TP versus TA).

### Participants

Participants were 600 Amazon Mechanical Turk (AMT) workers who were compensated US$1.00 for the 5–10-min experiment. There were 11 participants excluded for failing attention-check questions relating to the content of the stimulus video, leaving 589 participants (simultaneous TP = 139, simultaneous TA = 141, sequential TP = 161, sequential TA = 148) for the eventual analysis.

### Materials

This study employed a pool of 16 female lineup members, drawn from the Adelaide Lineup Database. This consists of a video and accompanying head-and-shoulders photographs taken front-on, at 90° side-on, and approximately at 45° for each of 194 persons. Only front-on photos were used in this study. In each video, the actor wears a black shirt T-shirt with a white University of Adelaide logo to remove the identifying potential of coloured clothing in the lineup phase. The scene opens with an actor (each of the 194 persons in turn) seated at a computer with their back to the camera. After a few seconds during which they type on the computer keyboard, the actor picks up a mobile phone placed on the table to their left and turns to face the camera while looking at the phone. The actor then stands and walks towards the camera while looking at the phone, glancing up briefly to the camera as they pass by. Each video is approximately 10–15 s in duration. An example video can be found at https://osf.io/p2hck/.

#### Stimulus pool selection process

In order to minimise the potential for stimulus effects, rather than a single target and set of foils, we used a pool of lineup members that could all act as both targets and foils. The starting point for selecting the pool members was similarity ratings previously collected for front-on photographs of 90 female faces in the Adelaide Lineup Database. AMT workers (*n* = 76) were compensated US$1.30 to rate 45 pairs of faces on a Likert scale from 0 (most similar) to 10 (least similar). Each participant rated a different subset of the possible face pairs to reduce participant burden and ensure timely collection of the data. The average number of ratings per similarity pair was 5.92, minimum 1, maximum 10. This resulted in a similarity matrix with each cell containing the mean rating of similarity between each pair of faces.

We first summed across each row of the similarity matrix, giving the mean similarity of a face relative to all other faces. Faces were then sorted from most similar to all others to least similar to all others. While this ordering served as a guide, we also identified a set of feature-based exclusion criteria, some of which related to distinctive non-biological features that appear in the photographs and others that related to constraints in terms of isolating a suitably large feature-matched subset from within the corpus. We excluded participants with nose rings or other obvious piercings, those wearing glasses, those who were not Caucasian in appearance, those with “unnaturally” dyed hair, e.g. blue hair, those with hair shorter than shoulder length and those with their hair pulled back. This resulted in a pool of 16 lineup female members of a similar ethnicity, skin tone, hair colour and hairstyle. One of the stimulus photographs required some editing to remove distinctive clothing features that were not obscured by the black T-shirt worn by all actors.

### Procedure

The entire procedure took place within AMT, with the experiment rendered on the participants’ web browsers. Participants were allocated to one of the four conditions on a round-robin basis. They were first questioned on their understanding of the task, being directed back to the instruction page if incorrect responses were recorded. They were then shown a video of a target randomly selected from the 16-member pool, before completing a visual search distractor task, similar in nature to a “Where’s Waldo/Wally”. Participants were then shown pre-lineup instructions corresponding to those in the U.S. National Deparment of Justice (1999) guidelines before viewing either a target present (TP) or target absent (TA) lineup presented simultaneously or sequentially, with the appropriate number of foils (5 for TP, 6 for TA) randomly selected from the remaining 15 members of the stimulus pool. The position of the target on TP lineups and the order of the foils on both TP and TA lineups was randomised.

In the simultaneous condition, participants could either identify a lineup item or choose a black silhouette to indicate that the target was not present in the lineup, after which they provided a confidence rating for their choice by typing a number from 0 to 100, where 0 was lowest confidence and 100 was highest confidence. In the sequential condition, participants were shown each lineup item individually with an option either to identify or to reject it. If the item was rejected, the next item in the sequence was shown. If a lineup item was identified, the procedure terminated and the participant was asked to provide a typed confidence estimate for their identification. If all lineup items were rejected, participants were informed that the lineup had been exhausted, indicating a rejection decision, and were asked for a typed confidence rating. Participants then answered follow-up questions about the clarity of the instructions and the difficulty of the task, and were given the opportunity to provide feedback.

### Analyses

We fit SDT-MAX and SDT-INT to the simultaneous data and SDT-SEQ to the sequential data, estimating seven parameters, *d*_*t*_, *s*_*t*_, and *c*_1_, *…*, *c*_5_, for each dataset. In Supplement 1 we provide annotated R code for fitting a multi-criteria, unequal variance version of SDT-MAX to simultaneous lineup data.

We tested our hypotheses using likelihood-ratio tests, comparing an unconstrained model to seven nested models where an equality constraint across the simultaneous and sequential data was imposed for one or the other parameter. We fit both conditions simultaneously, minimising *χ*^2^ for the overall fit. This allowed us to specify equality constraints across both conditions.

### Results and discussion

Table 4 shows the decision outcome frequencies for simultaneous and sequential lineups. The bin widths were set by collapsing over all conditions and partitioning the confidence judgements in to even-as-possible frequency quintiles. We used an alpha level of .05 for the model fits and hypothesis tests.

**Table 4:** Decision outcomes frequencies for simultaneous and sequential presentation

Simultaneous						
Confidence	100–91	90–81	80–66	65–51	50–0	Reject
TP – target ID	24	25	30	9	11	19
TP – foil ID	0	1	5	4	11	
TA – foil ID	4	11	25	16	24	61
Sequential						
Confidence	100–91	90–81	80–66	65–51	50–0	Reject
TP – target ID	32	22	21	13	6	41
TP – foil ID	0	3	7	9	7	
TA – foil ID	3	5	31	11	14	84

*TP* target present, *TA* target absent

#### Model fit performance and parameter estimates

Table 5 shows the recovered parameter values and fit statistics for SDT-MAX and SDT-INT fit to the simultaneous data and SDT-SEQ fit to the sequential data. For the simultaneous condition, both SDT-MAX and SDT-INT fit the data well. For the sequential condition, SDT-SEQ provided an adequate fit to the data. Table 5 shows that simultaneous and sequential *s*_*t*_ are similar when SDT-MAX is the simultaneous lineup model. This means that the *d*_*t*_ values for each presentation format are comparable estimates of underlying discriminability. In contrast, *s*_*t*_ is twice as large for simultaneous presentation compared to sequential presentation when SDT-INT is the simultaneous lineup model. In this case, the *d*_*t*_ values for each presentation format cannot be interpreted as directly comparable estimates of underlying discriminability. This is because, holding all else equal, increasing *s*_*t*_ increases the area of overlap between the target and foil distributions, reducing underlying discriminability.It is also evident from Table 5 that the decision criteria (*c*) estimated by SDT-INT are spread wider than those estimated by both SDT-MAX and SDT-SEQ. This is because they are scaled according to the detection decision variable for SDT-INT, the sum of the familiarity of all lineup items. Consequently, the decision criteria estimated by SDT-INT are not directly comparable to those estimated by SDT-SEQ (or SDT-MAX). In contrast, the decision variables of SDT-SEQ and SDT-MAX are both based on “untransformed” signal strengths and are therefore directly comparable.These difficulties in comparing the parameter estimates of SDT-INT to SDT-SEQ mean that SDT-INT is not well suited to testing our hypothesis. As a result, we employ SDT-MAX as the simultaneous lineup model and SDT-SEQ as the sequential lineup model in all subsequent analyses.

**Table 5:** Parameter estimates from fitting SDT-MAX and SDT-INT to the simultaneous data and SDT-SEQ to the sequential data from experiment 1

	Simultaneous		Sequential
	SDT-MAX	SDT-INT	SDT-SEQ
*d*_*t*_	1.83	2.56	1.89
*s*_*t*_	.94	2.02	1.12
*c*_*5*_	2.72	5.17	2.74
*c*_*4*_	2.20	3.41	2.27
*c*_*3*_	1.69	1.56	1.74
*c*_*2*_	1.49	.79	1.54
*c*_*1*_	1.16	−.54	1.41
*χ*^2^	13.44	12.19	15.39
*df*	8	8	8
*p*	.10	.14	.05

#### Underlying discriminability

Table 6 shows the results of the likelihood-ratio tests of the equality of each parameter between the simultaneous and sequential conditions as estimated by the SDT-MAX and SDT-SEQ models, respectively. The estimates of *d*_*t*_ and *s*_*t*_ did not differ significantly between the simultaneous and sequential conditions.

**Table 6:** Likelihood ratio tests comparing fits of unconstrained models to a series of constrained models where equality for each parameter is imposed across the simultaneous and sequential conditions

	*χ*^2^(1)	*p*
*d*_*t*_	.15	.70
*s*_*t*_	.87	.35
*c*_*5*_	.01	.91
*c*_*4*_	.28	.60
*c*_*3*_	.28	.60
*c*_*2*_	.48	.48
*c*_*1*_	10.54	< .01

Significant *p* values indicate that model fit significantly worsened when a parameter was constrained to be equal across the simultaneous and sequential conditions. For each unconstrained model, we fit SDT-SEQ to the sequential data and SDT-MAX to the simultaneous data. The unconstrained models had 16 degrees of freedom, fixing one parameter increases the degrees of freedom to 17, *χ*^2^(17) - *χ*^2^(16) = *χ*^2^(1), thus the *χ*^2^ tests above have one degree of freedom

The lack of a significant difference in underlying discriminability between simultaneous and sequential lineups is consistent with our previous re-analysis of the Palmer-Brewer database. It suggests that this result is not easily attributable to non-random target position in sequential lineups or the use of a designated innocent suspect selected to resemble the target to a greater extent than the foils. We also attempted to address the lack of statistical power in many of the studies in the Palmer-Brewer database. Despite increasing the number of participants compared, we did not observe a statistically significant difference in underlying discriminability. This suggests that if there is a simultaneous advantage, it is small and therefore difficult to detect. The effect size as measured by Hedge’s *g* for the difference between simultaneous and sequential underlying *d*_*t*_ is small, *g* = .06.

Additionally, our conclusion rests on the assumption that the SDT-MAX model is an appropriate model of the simultaneous lineup data. Recently, Wixted et al. (2018) proposed the *ensemble model* based on the idea of comparing diagnostic features.^2^ In this model, the item with the maximum familiarity (and potential target) is compared to the average familiarity of the remaining items. If this difference exceeds an evidential criterion, the potential target is identified, otherwise the lineup is rejected. We also fit this model to data from the simultaneous condition of experiment 1 and found that it provided an excellent fit, χ^2^(8) = 6.96, *p* = 0.54. However, we again found no statistically significant difference between its estimate of *d*_*t*_ and the estimate from the SDT-SEQ model, χ^2^(1) = 0.29, *p* = 0.59.

#### Response bias

Table 5 shows that estimates of decision criteria (*c*_2_, *…*, *c*_5_) are comparable between simultaneous and sequential lineups for each criterion except *c*_1_, which separates lineup identification and rejection decisions (the choose/no choose threshold). Table 5 shows that *c*_1_ was significantly larger in the sequential condition, supporting our hypothesis and conforming to previous literature (Carlson et al., 2016; Clark, 2012; Dobolyi & Dodson, 2013; Gronlund, Carlson, Dailey, & Goodsell, 2009; Meissner, Tredoux, Parker, & MacLin, 2005). Interestingly, having made this decision, the assignment of additional confidence levels did not differ between the two procedures.

#### Target distribution variance

Table 6 shows that estimates of target distribution variance (*s*_*t*_) did not differ between simultaneous and sequential presentation. The *s*_*t*_*v*alues displayed in Table 5 are also close to 1 for both presentation formats, implying that equal-variance models may account for these data. Constraining the models so that *s*_*t*_ *= s*_*s*_ *=* 1 did not significantly worsen the fit for SDT-MAX, *χ*^2^(1) = .28, *p* = .60, or SDT-SEQ, *χ*^2^(1) = .61, *p* = .43. This indicates that equal-variance models adequately capture these data, in contrast to long-standing findings of unequal target and lure distribution variance reported in the literature on basic recognition memory (Egan, 1958; Mickes et al., 2007) and in recent lineup research (Wilson et al., 2019; Wixted et al., 2018).

### Sequential position 1 compared to the simultaneous lineup

In addition to greater underlying discriminability in the simultaneous lineup, DFDT also predicts that underlying discriminability should increase over the course of the sequential lineup (Wixted & Mickes, 2014). The presentation of each new sequential lineup item provides an additional opportunity to isolate distinctive features uniquely shared by the target and the lineup items. Consistent with this, Wilson et al. (2019) identified greater underlying discriminability at sequential target positions 2–6 compared to position 1. This suggests that the difference in discriminability between sequential and simultaneous presentation should be greatest at sequential position 1 and should reduce over the course of the lineup. Because position 1 in a sequential lineup is equivalent to a single-item show up, this result is also consistent with the robust finding that the simultaneous lineup outperforms the single-suspect show up (Gronlund et al., 2012; Neuschatz et al., 2016; Wooten et al., 2020).

When comparing underlying discriminability between simultaneous and sequential presentation, differences between the simultaneous lineup and each sequential position are aggregated. Fully randomising the position of the target, as in our experiment, may have reduced the average simultaneous advantage, which may explain why we failed to find one. To investigate this possibility, we compared underlying discriminability between sequential position 1 and the simultaneous lineup.

#### Data

Table 7 shows the frequency counts for sequential serial position 1 (i.e. show up) data and the simultaneous lineup. Because of the comparatively small number of TP trials in sequential position 1, it was not possible to classify the data further by confidence level. In order to treat responses to sequential position 1 as a show up, we reclassified participants’ responses as follows. A TP_1_ show-up trial occurred when the first sequential lineup item was the target. A TA_1_ show-up trial occurred when the first sequential lineup item was a foil. Note that this includes those participants who encountered the target at a later serial position in the lineup as well as those who never saw a target.

**Table 7:** Decision outcomes frequencies for sequential serial position one, treated as a showup, and the simultaneous lineup

Showup (Sequential Serial Position One)		
	Identify	Reject
TP_1_ – Target ID	15	13
TA_1_ – Foil ID	19	262
Simultaneous Lineup		
	Identify	Reject
TP – Target ID	99	19
TP – Foil ID	21	
TA – Foil ID	80	61

#### Model fits and results

We used an equal variance (EVSD) model of the yes/no task to estimate show up *d*_*t*_ and *c* and SDT-MAX to estimate simultaneous *d*_*t*_ and *c*. As previously, we conducted likelihood ratio tests comparing the overall fit of an unconstrained model fit to each dataset simultaneously, to various constrained models where one parameter was set to be equal across the two sets of data.

We fit the EVSD model to the show-up data. In this case, it has an analytic solution given by, *d*_*t*_ = Φ^− 1^(H) – Φ^− 1^(F) and *c* = Φ^− 1^(1 – F), where H is the Target ID rate, F is the TA Foil ID rate and Φ^− 1^ is the inverse normal cumulative distribution function. Because there are no degrees of freedom, this model necessarily fits perfectly. The estimated parameter values were, *d*_*t*_ = 1.58 and *c* = 1.49. We fit the SDT-MAX to the simultaneous data with the constraint that *s*_*t*_ = 1. It fit these data well, *χ*^2^(1) = 2.54, *p* = .11, with estimated parameter values, *d*_*t*_ = 1.98 and *c* = 1.18. Although underlying discriminability appeared to be greater for the simultaneous lineup, this difference was not significant, *χ*^2^(1) = 1.87, *p* = .17. Responding was significantly more conservative for sequential position 1, *χ*^2^(1) = 5.79, *p* < .05, consistent with previous findings at the aggregate level.

Despite previous studies that have reported a simultaneous advantage in underlying discriminability over show ups (e.g. Neuschatz et al., 2016) we failed to observe a similar effect in our data. Because the experiment was not designed with this analysis in mind, the number of participants in the TP_1_ was relatively small (*N* = 28) which means that the analysis may not have sufficient statistical power. Nevertheless, it is possible to conclude that if there is an advantage for simultaneous presentation it is likely to be a relatively small effect.

## Re-analysis of simultaneous versus sequential studies conducted since Palmer and Brewer (2012)

We failed to find an underlying discriminability advantage for the simultaneous lineup compared to the sequential lineup in a corpus of studies published prior to Steblay and Phillips (2011) and in our own experimental data. However, it is possible that such an effect occurs in studies published after Steblay and Phillips (2011), particularly those that report an empirical discriminability advantage for simultaneous presentation (e.g. Mickes et al., 2012). We conducted a literature search for studies published since 2011 that compared photographic simultaneous and stopping-rule sequential lineups. We isolated studies that reported results in such a way that we could extract the cell frequencies required to fit the SDT-MAX and SDT-SEQ models. Seven simultaneous versus stopping-rule sequential lineup studies published since 2011 met our criteria; Carlson and Carlson (2014), Carlson et al. (2016), Flowe et al. (2016), Pica and Pozzulo (2017), Pozzulo, Dempsey, and Pettalia (2013), Pozzulo, Reed, Pettalia, and Dempsey (2016) and Sučić et al. (2015). Additionally, we requested the data from Mickes et al. (2012), from which we were able to extract the required cell frequencies for experiment 1a, but not experiments 1b or 2. This new corpus of eight studies (total *N* = 6453, simultaneous *n* = 2803, sequential *n* = 3650) provides more power to detect a simultaneous advantage in underlying discriminability than the Palmer and Brewer corpus (total *N* = 3871, simultaneous *n* = 1952, sequential *n* = 1919).

### Method

As per our analysis of the Palmer and Brewer corpus, we estimated *d*_*t*_*, c* and, where relevant, *d*_*s*_ for each study by fitting SDT-MAX to the simultaneous data and SDT-SEQ to the sequential data. We then calculated mean discriminability (*d*_*t*_ – *d*_*s*_) and response bias (*c*) weighted by sample size for simultaneous and sequential presentation. For most of the studies, we estimated parameters separately for each experimental condition, rather than collapsing over conditions other than presentation format. This led to thirteen simultaneous versus sequential datasets from the eight studies. For Carlson and Carlson (2014) and Carlson et al. (2016), we collapsed the sequential target position-2 and target position-5 conditions, specifying that the target could only appear at these two positions when fitting SDT-SEQ. For Pozzulo et al. (2013) we collapsed the adolescent and adult age conditions because the original study reported no effect of age on decision performance.

### Results

Model-fit statistics and parameter values for each dataset are available in Table S2. SDT-MAX fit 12 of 13 simultaneous datasets at α = .05, failing to fit the backloaded simultaneous condition of Carlson et al. (2016). SDT-SEQ fit 10 of 13 sequential datasets at α = .05, failing to fit the sequential data from Sučić et al. (2015), the sequential weapon present plus distinctive feature condition from Carlson and Carlson (2014) and the sequential data from Pozzulo et al. (2013). Table 8 shows the mean and standard deviations for discriminability and response bias (*c*) for simultaneous and sequential presentation, weighted by sample size. The Welch two-sample weighted *t* test indicated no significant difference in mean weighted discriminability, *t* (21.43) = 1.14, *p* = .27 or mean weighted response bias*, t* (20.72) = 0.08, *p* = .94, between presentation formats. As for the Palmer and Brewer corpus and our experiment, this does not support the hypothesis that underlying discriminability is greater for simultaneous presentation. Unlike our previous analyses, the hypothesis that responses are more conservative in the sequential procedure was not supported.

**Table 8:** Mean parameter values weighted by sample size from fits of SDT-MAX to simultaneous lineup data and SDT-SEQ to sequential lineup data from a corpus of eight studies published since 2011

Format	Source	Parameter			
		*discriminability*		*c*	
		*μ*_*w*_	*σ*_*w*_	*μ*_*w*_	*σ*_*w*_
Simultaneous	SDT-MAX	1.23	.54	1.09	.21
Sequential	SDT-SEQ	1.02	.38	1.09	.32

## General discussion

The present study sought to compare performance between the simultaneous lineup and sequential stopping-rule lineup in order to test the central prediction of the diagnostic feature detection hypothesis; that underlying discriminability is greater when lineups are administered simultaneously rather than sequentially (Wixted & Mickes, 2014). As structural differences between the procedures affect the shape of the corresponding ROCs, a difference in empirical discriminability between simultaneous and sequential presentation does not necessarily indicate a difference in underlying discriminability. In order to measure underlying discriminability, it is necessary to characterise the data in terms of an appropriate model. Accordingly, we developed a novel signal detection model that captures the structure of the sequential lineup task, SDT-SEQ, and contrasted this with models of the simultaneous lineup task, SDT-MAX and SDT-INT (as well as the ensemble model).

We first fit SDT-MAX, SDT-INT and SDT-SEQ to the Palmer and Brewer (2012) database comprising a set of earlier studies that directly compared simultaneous and sequential stopping-rule presentations. While we identified and corrected a number of methodological shortcomings in the original study, the conclusions that we reached were the same. First, we found no systematic difference in underlying discriminability between the two kinds of lineup (measured by the parameter, *d*_*t*_, or *d*_*t*_*– d*_*s*_ where relevant). Second, we found a shift to a more conservative response bias in sequential lineups. As the studies in the database did not collect or report post-decision confidence estimates, we were unable to estimate all the parameters specified in our models, leaving more nuanced aspects of the simultaneous versus sequential presentation question unexplored. Most studies also had relatively small numbers of participants and so lacked statistical power to detect a small effect, they selected designated innocent suspects designed to resemble the target and they did not randomise the position of the target in sequential lineups. For this reason, we conducted a more powerful experiment that elicited multiple confidence judgements, did not employ a designated innocent suspect and randomised the position of the target on sequential lineups. We found no significant difference in underlying discriminability and more conservative responding for the sequential lineup, consistent with the Palmer and Brewer re-analysis. Finally, we analysed a corpus of data containing eight recent lineup studies that compared simultaneous and sequential presentation. The results were consistent with the previous findings in that there was no significant difference in underlying discriminability, but we did not find more conservative response bias for sequential presentation.

**Fig. 5:**
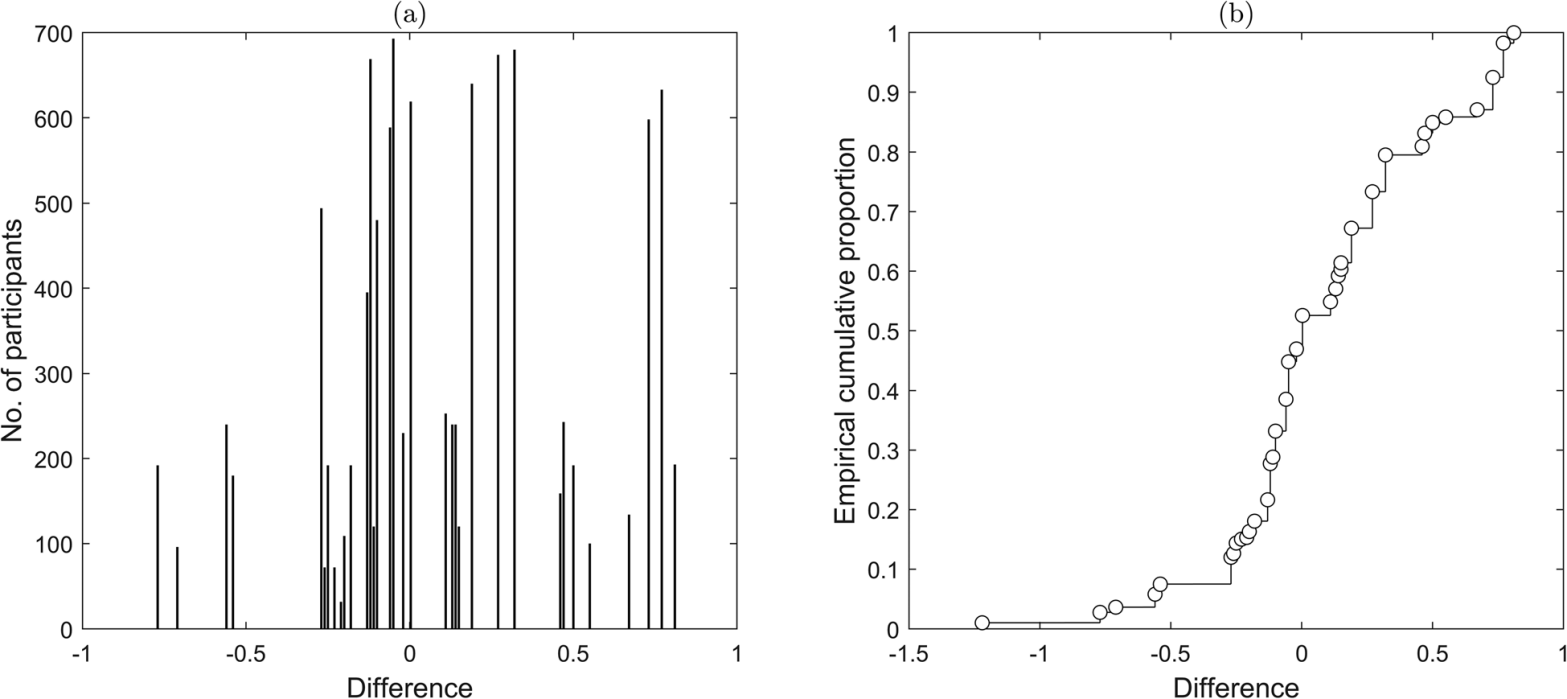
Summary plot of the observed difference in underlying discriminability between simultaneous and sequential presentation. **a** Histogram plot. Each bar corresponds to an observed difference. The length of the bar equals the number of participants on which the estimate is based. **b** Empirical cumulative distribution plot. The same data plotted as an ogive

Our analyses provide estimates of the difference in underlying discriminability between simultaneous and sequential lineups across a total of 36 separate studies or conditions within studies. While many features of these studies (e.g. lineup size, target position, presence of a designated suspect, backloading) vary considerably, each provides a point estimate of the difference in underlying discriminability. These estimates are plotted in Fig. 5 panel A weighted by the number of participants and in panel B as a cumulative proportion ogive. Panel A can be viewed as a “group-based” histogram in which each participant is assigned the difference estimate calculated for their group as a whole. Each vertical bar is centred on a given estimate and the length of the bar corresponds to the total number of participants in the group. The total number of participants across all the studies is 10,913. According to these data, the overall weighted mean difference is 0.09, indicating a slight advantage for simultaneous lineups. The same data are plotted in panel B as a cumulative proportion ogive. From this, it is possible to determine that the median difference is 0.03, the 5th percentile is − 0.56 and the 95th percentile is 0.77. Thus, in the studies we have analysed, approximately 50% of participants can be presumed to have shown a simultaneous advantage in underlying discriminability while the remaining 50% show the opposite. Overall, this means that although some more recent studies have observed a simultaneous advantage in underlying discriminability, the evidence to date taken as a whole suggests that this effect is close to zero.

### Diagnostic feature detection theory

Our results are not consistent with a key prediction of diagnostic feature detection theory (DFDT), that the greater opportunity to compare lineup items in the simultaneous lineup should improve underlying discriminability compared to the sequential lineup. However, the lack of an easily detected difference in underlying discriminability between simultaneous and sequential lineups does not necessarily militate against the processes proposed by the DFDT. All things being equal, it is possible that the greater detectability of diagnostic features in simultaneous lineups may lead to a performance advantage. However, this is a critical caveat - there may be other differences between the procedures that serve to counteract this effect. One obvious difference is the size of the choice set. In a simultaneous lineup, the target (if present) is one of several alternatives while in a sequential lineup, on each trial only a single item is presented. It is well-known that the probability of correct target detection declines with the increasing size of the choice set (Swets, 1959). On the other hand, it is possible that sequential presentation may induce retroactive interference through re-encoding of lineup items into memory. This would be expected to have a greater impact on items appearing later in the sequence which is suggested by the finding reported by Wilson et al. (2019) that underlying discriminability may increase over the course of the sequential lineup, at least after position 1. The point is that because the two procedures have different characteristics, it is likely they induce a range of effects on memory which, in the cases we have so far examined, more or less cancel out. Diagnostic feature detection may well occur but its effects on memory may be counteracted by other differences.

The foregoing analysis suggests that if relevant differences between simultaneous and sequential lineups could be reduced then the effects of diagnostic feature detection may be revealed. A recent study by Colloff and Wixted (2020) bears on this issue. They compared a standard show up in which only the suspect was presented with a novel *simultaneous show up* in which the suspect was presented along with five fillers, none of which could be identified as the target. Based on ROC analysis, they found that the opportunity to compare the suspect to other similar faces in the simultaneous show-up procedure improved empirical discriminability. Because the structural characteristics of the standard and simultaneous lineups are essentially the same - both require a decision to be made about a single item - the difference in empirical discriminability suggests a corresponding difference in underlying discriminability. If so, then the results reveal the kind of advantage predicted by the DFDT.

### The UK lineup procedure

In a series of studies, Seale-Carlisle and colleagues have investigated the empirical and underlying discriminability of the UK (or Police and Criminal Evidence (PACE)) lineup procedure (Seale-Carlisle et al., 2019; Seale-Carlisle & Mickes, 2016; Wixted et al., 2018). This procedure is conducted in accordance with the UK Police and Criminal Evidence guidelines (Police and Criminal Evidence Act 1984, Code D, 2017). It differs in important ways from the stopping rule sequential lineup. First, witnesses see short videos of each lineup member rotating through a head-and-shoulders profile rather than a static photo. Second, witnesses must view two full laps of the lineup procedure before making a decision, i.e. the lineup does not have a stopping rule, and may return to any item as many times as they wish before making their decision. In addition, the UK lineup contains nine items rather than six, as is common in other jurisdictions.

Seale-Carlisle and Mickes (2016) found that the UK lineup procedure had lower empirical discriminability based on ROC analysis than a comparable simultaneous lineup. Seale-Carlisle et al. (2019) conducted a series of experiments to try to isolate which aspects of the UK procedure were responsible for this difference. They also examined underlying discriminability by fitting the ensemble model to different versions of the UK lineup. They concluded that the crucial feature that impaired relative performance in the UK lineup was the sequential presentation format. This was identified in one experiment (experiment 1) and partially verified in a second experiment (experiment 5). That is, both experiments found a difference in empirical discriminability based on measurement of the area under the ROC curve, but although there was a significant difference in underlying discriminability in the first experiment, this was not replicated in the second.

The results of Seale-Carlisle et al. (2019) are, to our knowledge, the only example of a significant simultaneous lineup advantage in underlying discriminability. Because there is no stopping rule, witnesses make their decision after having viewed all the lineup items. Therefore, in terms of the task demands, the UK lineup functions as a kind of simultaneous lineup in which viewing of items is constrained to be sequential. The decrement in underlying discriminability identified by Seale-Carlisle et al. appears to be a consequence of this feature. However, our previous analyses suggest that it may not be a consequence of sequential presentation per se. These show that sequential presentation with a stopping rule does not significantly impair underlying discriminability. The difference must lie elsewhere. One possibility is that the UK procedure places additional memory demands on witnesses who must encode information about the members in the lineup, such as their facial features and lineup position, for a future identification decision. This may lead to the build-up of retroactive interference between test items and target memory (Dewar, Cowan, & Sala, 2007; Sosic-Vasic, Hille, Kröner, Spitzer, & Kornmeier, 2018; Wickelgren, 1966). In contrast, the presence of a stopping-rule reduces memory demands because once a decision is made, the features of the current lineup item can be immediately forgotten.

Consistent with previous studies (Carlson et al., 2016; Clark, 2012; Dobolyi & Dodson, 2013; Gronlund et al., 2009; Meissner et al., 2005; Palmer & Brewer, 2012), we found that sequential presentation led to more conservative responding. This conforms to the original intention behind the introduction of sequential lineups, to reduce false alarms.

### Conclusions

This study introduced a new model of the sequential lineup task, SDT-SEQ, and in conjunction with models of the simultaneous lineup task, SDT-MAX and SDT-INT, tested a key prediction of the diagnostic feature detection theory that underlying discriminability should be greater in a simultaneous lineup. In both our re-analysis of the Palmer and Brewer (2012) database and data from eight recently published studies, in addition to the results of a new experiment, we did not find evidence consistent with this prediction. This suggests that if the effect exists, it may be counteracted by other effects associated with differences between the two kinds of task. Further research is required to determine the conditions under which comparing features across lineup items improves memory, the limits of such an effect, and the extent to which it is affected by structural aspects of different lineup tasks.

## Supplementary information

**Additional file 1.** Model Equations.

**Additional file 2.** Model Simulations and Cross Fits.

**Additional file 3: Table S1.***d*_*t*_*, d*_*s*_*, c* and *C* for each dataset in the Palmer and Brewer (2012) corpus, estimated by the relevant models for each presentation format. **Table S2.** Fit statistics and *d*_*t*_*, d*_*s*_ and *c* for each dataset in the post-2011 corpus, estimated by SDT-MAX and SDT-SEQ for simultaneous and sequential presentation respectively.

**Additional file 4: Supplement 1.** Fitting SDT-MAX to Simultaneous Lineup Data.

## Data Availability

The experimental data from this project is available on the Open Science Framework repository at https://osf.io/769hy/.

## Footnotes

1 We differentiate innocent suspects from known-innocent foils here because, in some studies, innocent suspects are selected to bear a closer resemblance to the target than the foils. This means that innocent suspect should be modelled as being drawn from a distribution with a different mean to that of the foil distribution.

2 Full details of the ensemble model available in (Wixted et al., 2018).
