# Supplementary material for: Do sequential lineups impair underlying discriminability?
Source: Cogn Res Princ Implic. 2020 Aug 4;5:35. doi: 10.1186/s41235-020-00234-5 (PMC7403381; doi:10.1186/s41235-020-00234-5)
Supplement: Supplementary file 1 — Additional file 1. Model Equations. [file 41235_2020_234_MOESM1_ESM.docx]

# Additional file 1

# Model Equations

We suppose that each witness is presented with a lineup consisting of *n* items. Let $\left[n\right]=\left\{1,\ldots,n\right\}$ be the set of item positions in the lineup and, for a given witness, let $X=\left\{x_{i}:i\in\left[n\right]\right\}$ be the set of familiarity values of the corresponding items. In a target present (TP) lineup, there is one *target* and $n-1$ *foils*. Similarly, in a target absent (TA) lineup, there may be one designated *suspect* and $n-1$ foils. Alternatively, if there is no designated suspect then the lineup consists only of foils.

We assume that each $x_{i}$ is a value of a continuous random variable that defines a probability distribution for that item. We also assume that the values of each random variable are independently and identically distributed. Let $T\left(\cdot\right)$ and $t\left(\cdot\right)$ be the cdf and pdf of the target distribution and let $S\left(\cdot\right)$ and $s\left(\cdot\right)$ be the cdf and pdf of the designated innocent suspect distribution. We assume that the random variables corresponding to foils all have the same distribution. Accordingly, let $F\left(\cdot\right)$ and $f\left(\cdot\right)$ be the cdf and pdf, respectively, of the common foil distribution.

The witness identification task can be notionally partitioned into a *detection task* and an *identification task* (Duncan, 2006). The aim of the detection task is to determine if the lineup contains a target. The aim of the identification task is to determine which lineup item is the target. Accordingly, we define three events of interest. In a TP lineup, a *target detection* (TD) occurs when a witness identifies any item as the target and a *target identification* (TID) occurs when the item so identified is in fact the target. In a TA lineup, a *false alarm* (FA) occurs when a witness identifies any item as the target and a *suspect identification* (SID) occurs when the item so identified is the designated innocent suspect (if present). From these events, additional events can be defined. A *miss* occurs when there is no target detection to a TP lineup; a *correct rejection* occurs if there is no false alarm to a TA lineup; a *foil identification* (FID) occurs if there is target detection but no correct identification in a TP lineup or if there is a false alarm but no suspect identification in a TA lineup.

When a witness identifies a lineup item, they assign to it a confidence level according to a set of *k* decision criteria, $C=\left\{c_{i}:i\in[k]\right\}$. For a given $c\in C$, we define $P_{TID}\left(c\right)$, $P_{TD}\left(c\right)$, $P_{SID}\left(c\right)$ and $P_{FA}\left(c\right)$ as the proportions of target identifications, target detections, suspect identifications, and false alarms, respectively, assigned a level of confidence at least as great as *c*. We note that $P_{FID}\left(c\right)=P_{FA}\left(c\right)-P_{SID}\left(c\right)$ for TA lineups and$P_{FID}\left(c\right)=P_{TD}\left(c\right)-P_{TID}\left(c\right)$ for TP lineups. If there is no designated suspect then $P_{SID}\left(c\right)=P_{FA}\left(c\right)/n$.

We assume that the elements of *C* are ordered $c_{1}<c_{2}<\ldots<c_{k}$. The expected proportion of target identifications in confidence band *i* is then equal to $P_{TD}\left(c_{i}\right)-P_{D}\left(c_{i+1}\right)$ for $i<k$ and equal to $P_{TD}\left(c_{i}\right)$ for $i=k$. The proportion of non-identifications (i.e., misses) is equal to $1-P_{TD}\left(c_{1}\right)$. Similar considerations apply to false alarms and correct rejections as well as to target and suspect identifications although these cases, these are undefined if no identification is made.

## SDT-MAX

SDT-MAX is a simultaneous lineup model that implements the following decision rule. Let $m\in\left[n\right]$ such that $x_{m}=\max\left(X\right)$. Then if $x_{m}\geq c_{1}$, identify item *m* as the target at confidence level, $c=\max\left(\left\{c\in C:c\leq x_{m}\right\}\right)$, otherwise reject the lineup. The general equations for this model are as follows.

For a TP lineup,

$$P_{TD}\left(c\right)=1-T\left(c\right)F\left(c\right)^{n-1}.$$

Let $t\in\left[n\right]$ be the position of the target item in a TP lineup. Then $P_{TID}\left(c\right)$ is the joint probability that $m=t$ and$x_{m}\geq c$. The probability that $m=t$is

$\text{Pr}\left(m=t\right)=\int_{-\infty}^{\infty} t\left(x\right)F\left(x\right)^{n-1}dx$ . (1)

The additional requirement that $x_{m}\geq c$ gives the equation,

$P_{TID}\left(c\right)=\int_{c}^{\infty} t\left(x\right) F\left(x\right)^{n-1}dx$.

Similarly, for a TA lineup with a designated innocent suspect,

$$P_{FA}\left(c\right)=1-S(c)F\left(c\right)^{n-1}$$

and

$P_{SID}\left(c\right)=\int_{c}^{\infty} s\left(x\right) F\left(x\right)^{n-1}dx$.

If there is no designated innocent suspect then

$$P_{FA}(c)=1-F(c)^{n}$$

and

$$P_{SID}\left(c\right)=P_{FA}\left(c\right)/n.$$

**Normal distribution implementation.** In order to fit this and other models we present, it is necessary to specify the forms of the different probability distributions. Following standard practice, we assume that they are normal. In this and following sections, let be the normal pdf with mean $\mu$ and standard deviation $\sigma$, evaluated at , and let be the corresponding normal cdf. Let$\mu_{t}$and $\sigma_{t}$ be the mean and standard deviation of the target distribution and let$\mu_{s}$and $\sigma_{s}$ be the mean and standard deviation of the innocent suspect distribution. Without loss of generality, the mean and standard deviation of the foil distribution is zero and one, respectively. In this case, we call and $\Phi(x;0,1)$ the standard normal cdf and pdf respectively, and write and $\Phi(x;0,1)=\Phi(x)$.

Substituting for $t(x)$,for $s(x)$and for $f(x)$, we derive the following equations for SDT-MAX:

If there is no designated innocent suspect then

## SDT-INT

The SDT-INT model is a simultaneous lineup model that implements the decision rule: if $\text{sum}\left(X\right)\geq c_{1}$ then choose lineup item *m* at confidence level $c=\max\left(\left\{c\in C:c\leq\text{sum}\left(X\right)\right\}\right)$, otherwise reject the lineup. The general equations for this model are as follows:

For a TP lineup,

$P_{TD}\left(c\right)=\int_{-\infty}^{\infty} t*f^{*\left(n-1\right)}(x)dx$,

where $t*f$ is the convolution of the density functions *t* and *f*, and $f^{*n}$ is the convolution of *f* with itself *n* times. That is, $f*f=f^{*2}$. Then $P_{TID}\left(c\right)$ is the joint probability that $\text{sum}\left(X\right)\geq c$ and $m=t$. This can be expressed as the following product,

$$P_{TID}\left(c\right)=\text{Pr}\left(\text{sum}(X)\geq c|m=t\right)\cdot\text{Pr}\left(m=t\right)$$

where $\text{Pr}\left(m=t\right)$ is given by Equation 1 above. We now require an equation for the conditional probability $\text{Pr}\left(\text{sum}\left(X\right)\geq c|m=t\right)$.

Let $X\backslash\left\{\left.x_{m}\right\}\right.=\left\{x_{i}\in X:i\neq m\right\}$be the set of memory strengths of the $n-1$items, excluding the maximum and let $Y=\text{sum}\left(X\backslash\left\{\left.x_{m}\right\}\right.\right)$. Then

$\text{Pr}\left(\text{sum}\left(X\right)\geq c\right)=\text{Pr}\left(Y\geq c-x_{m}\right)$.

Because $Y$is the sum of $n-1$ random variables, its pdf is equal to the convolution of the component pdfs, truncated at the upper limit of $x_{m}$. Let $f_{x}$be the distribution $f$truncated at $x$. That is,

$$f_{x}\left(u\right):=\left\{\begin{matrix} \frac{f\left(u\right)}{F\left(x\right)} & u\leq x, \\ 0 & u>x \end{matrix}\right.$$

Suppose $m=t$. Then,

$$\text{Pr}\left(Y\geq c-x\right)=\int_{c-x}^{\infty} f_{x}^{*(n-1)}\left(u\right)du$$

and hence

Similarly, for a TA lineup with a designated innocent suspect,

If there is no designated innocent suspect,

$$P_{FA}(c)=\int_{c}^{\infty} f^{*n}dx.$$

**Gaussian Implementation.** Because the convolution of two more Gaussian distributions is Gaussian, it follows that:

$$P_{TD}(c)=1-\Phi\left(c;\mu_{t},\sqrt{n-1+\sigma_{t}^{2}}\right).$$

Let be the standard normal pdf truncated at the upper limit $x$and let $\mu_{x}$and $\sigma_{x}^{2}$ be its mean and variance, respectively. It is known that

Because the memory strengths of the foils are independent, the mean and variance of the convolution of $n-1$ truncated distributions is $\mu_{x}$and $\sigma_{x}^{2}$ each multiplied by $n-1$. By the central limit theorem, this convolution is approximately normal. Therefore,

$$\text{Pr}\left(Y\geq c-x\right)\approx1-\Phi\left(c-x;(n-1)\mu_{x},\sqrt{n-1}\sigma_{x}\right)$$

and

Similarly, if there is a designated innocent suspect

If there is no designated innocent suspect then

$$\begin{matrix} P_{FA}\left(c\right)=1-\Phi\left(c;0,\sqrt{n}\right) \\ P_{SID}\left(c\right)=\frac{1}{n}\left(1-\Phi\left(c;0,\sqrt{n}\right)\right). \end{matrix}$$

## SDT-SEQ

The SDT-SEQ model is a sequential lineup model that implements the following decision rule. Let $K=\left\{i\in\left[n\right]:x_{i}\geq c_{1}\right\}$. That is, *K* is the set of positions of those items with memory strengths large enough to be identified. Then, if *K* is not empty choose lineup item $m=\min\left(K\right)$ at confidence level, $c=\max\left(\left\{c\in C:c\leq x_{m}\right\}\right)$, otherwise reject the lineup.

We start by deriving an equation for $P_{TID}\left(c\right)$ and *mutatis mutandis* for $P_{SID}\left(c\right)$. If the first lineup item is the target then $P_{TID}\left(c\right)=1-T\left(c\right)$. If the second item is the target then $P_{TID}\left(c\right)=\left(1-T\left(c\right)\right)F\left(c_{1}\right)$, and so on. In general, if the target is at position *t* then the probability of target identification is equal to the joint probability of identifying the target and not identifying an item at any preceding position. Let $p_{i}$ be the probability that item *i* is the target. Then

$P_{TID}\left(c\right)=\left(1-T\left(c\right)\right)\sum_{i=1}^{n} p_{i}F\left(c_{1}\right)^{i-1}$ .

The equation for $P_{SID}\left(c\right)$ is directly analogous. In this case, let $q_{i}$ be the probability that item *i* is the designated innocent suspect. Then

$P_{SID}\left(c\right)=\left(1-S\left(c\right)\right)\sum_{i=1}^{n} q_{i}F\left(c_{1}\right)^{i-1}$ .

The equations for $P_{TD}\left(c\right)$ and $P_{FA}\left(c\right)$ are more complex. In a sequential lineup, target detection occurs whenever the witness identifies any item in a TP lineup, whether it is the target or not. Suppose item *t* is the target and let $P_{TD}\left(c\right)_{t}$ be the probability of target detection in the corresponding lineup. This is the sum of three probabilities; $P_{TD}\left(c\right)_{i<t}$, the probability of identifying a preceding item as the target, $P_{TD}\left(c\right)_{i=t}$ the probability of correctly identifying the target at position *t*, and $P_{TD}\left(c\right)_{i>t}$, the probability of identifying a following item as the target. Then

$$\begin{matrix} P_{TD}\left(c\right)_{i<t}=\left(1-F\left(c\right)\right)\sum_{i=1}^{t-1} F\left(c_{1}\right)^{i-1} \\ P_{TD}\left(c\right)_{i=t}=\left(1-T\left(c\right)\right)F\left(c_{1}\right)^{t-1} \\ P_{TD}\left(c\right)_{i>t}=T\left(c\right)\left(1-F\left(c\right)\right)\sum_{i=t+1}^{n} F\left(c_{1}\right)^{i-2} \end{matrix}$$

with $P_{TD}\left(c\right)_{i<1}=0$ and $P_{TD}\left(c\right)_{i>n}=0$. It follows that $P_{TD}\left(c\right)_{t}=P_{TD}\left(c\right)_{i<t}+P_{TD}\left(c\right)_{i=t}+P_{TD}\left(c\right)_{i>t}$ and

$P_{TD}\left(c\right)=\sum_{i=1}^{n} p_{i}P_{TD}\left(c\right)_{i}$ .

By analogy, for a TA lineup with the designated innocent suspect in position *s*,

$$\begin{matrix} P_{FA}\left(c\right)_{i<s}=\left(1-F\left(c\right)\right)\sum_{i=1}^{s-1} F\left(c_{1}\right)^{i-1} \\ P_{FA}\left(c\right)_{i=s}=\left(1-S\left(c\right)\right)F\left(c_{1}\right)^{s-1} \\ P_{FA}\left(c\right)_{i>s}=S\left(c\right)\left(1-F\left(c\right)\right)\sum_{i=s+1}^{n} F\left(c_{1}\right)^{i-2}. \end{matrix}$$

Hence, $P_{FA}\left(c\right)_{s}=P_{FA}\left(c\right)_{i<s}+P_{FA}\left(c\right)_{i=s}+P_{FA}\left(c\right)_{i>s}$ and, if there is a designated suspect,

$P_{FA}\left(c\right)=\sum_{i=1}^{n} q_{i}P_{FA}\left(c\right)_{i}$ ,

otherwise,

$$P_{FA}\left(c\right)=\left(1-F\left(c\right)\right)\sum_{i=1}^{n} F\left(c_{1}\right)^{i-1}.$$

**Normal distribution implementation.** To implement the above as normal distributions, the following substitutions are made:

$$\begin{matrix} T\left(c\right)=\Phi\left(c;\mu_{t},\sigma_{t}\right) \\ S\left(c\right)=\Phi\left(c;\mu_{s},\sigma_{s}\right) \\ F\left(c\right)=\Phi\left(c\right). \end{matrix}$$
