# Supplementary material for: Do sequential lineups impair underlying discriminability?
Source: Cogn Res Princ Implic. 2020 Aug 4;5:35. doi: 10.1186/s41235-020-00234-5 (PMC7403381; doi:10.1186/s41235-020-00234-5)
Supplement: Supplementary file 2 — Additional file 2. Model Simulations and Cross Fits. [file 41235_2020_234_MOESM2_ESM.docx]

# Additional file 2

# Model Simulations and Cross Fits

We first generated random parameter values for *d_t_*, *s*, and five criteria (*c*), matching the seven-parameter model used in subsequent fits to our experimental data. Parameters were sampled from uniform distributions with ranges taken from fits to the Palmer and Brewer (2012) database for *d_t_* [0.3 – 2.6] and *c* [0.4 – 2.2] and from Wixted, Vul, Mickes, and Wilson (2018) and Wilson, Donnelly, Christenfeld, and Wixted (2019) and the subsequent fits to our experimental data for *s* [0.6 – 1.2]. We generated 100 sets of parameter values, which we used to simulate 100 datasets according to each model. The likelihood functions for generating predicted data according to each model are available in Appendix A. The models generate predicted proportions, not frequencies, but frequencies are required to fit the models to the data. In order to avoid any issues with low cell counts, we assumed 10000 TP and 10000 TA lineups for each dataset, multiplying the predicted TP/TA decision rates from the model by these amounts. Each model was then fit to the 100 datasets it generated, in addition to the 100 datasets generated by the other models.

**Results**

Table 1 and Table 2 below show the correlations between the parameter values of *d_t_* and *s* for all cross fits. In general, correlations are good when the models are fit to their own data, although SDT-SEQ, and to some extent SDT-INT, have problems with outliers when fit to their own data, likely caused by the model falling in to local minima in the parameter space. When these outliers are excluded, recovery improves substantially, as evident on the main diagonal of Figures 2 and 3. Figure 1 shows mean χ^2^ for each model fit to its own data and that of the other models. It is evident that the models tend to fit their own data better than the data generated by the other models. Figures 2 - 8 show scatterplots of the generating and recovered parameter values for *d_t_*, *s* and *c_1_ – c_5_* respectively. Criterion recovery is generally good when the models are fit to their own data, with the exception of some outliers for SDT-SEQ and SDT-INT when fit to their own data.

Table 1.

*Correlation between generating and recovered d_t_ for each cross fit*

|  |  | Fitting Model | | |
| --- | --- | --- | --- | --- |
| Generating Model |  | SDT-SEQ | SDT-MAX | SDT-INT |
|  | SDT-SEQ | .53 | .31 | .56 |
|  | SDT-MAX | .47 | 1.00 | .98 |
|  | SDT-INT | .97 | .99 | .99 |

Table 2.

|  |  | Fitting Model | | |
| --- | --- | --- | --- | --- |
| Generating Model |  | SDT-SEQ | SDT-MAX | SDT-INT |
|  | SDT-SEQ | .53 | .23 | .21 |
|  | SDT-MAX | .60 | 1.00 | .38 |
|  | SDT-INT | .29 | .59 | .67 |

*Correlation between generating and recovered s for each cross fit*

*
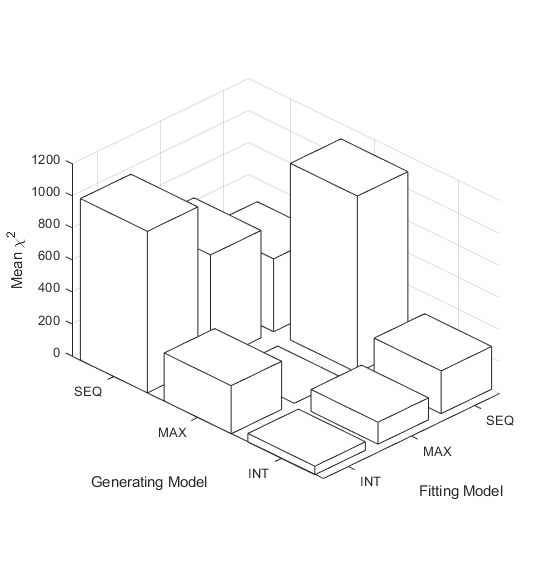
*

*Figure 1.* Mean χ^2^ values for each model fit to its own simulated data and cross fit to the data generated by the other models.


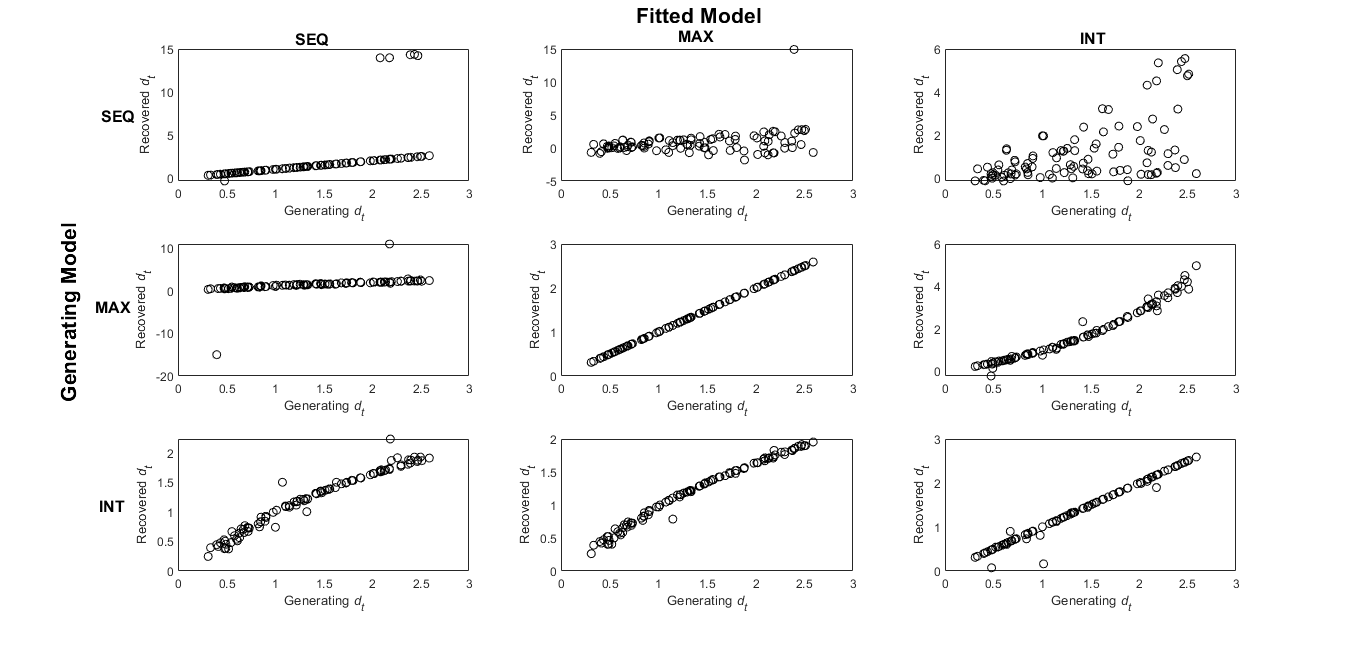


*Figure 2.* Scatter plots for *d_t_* cross fits


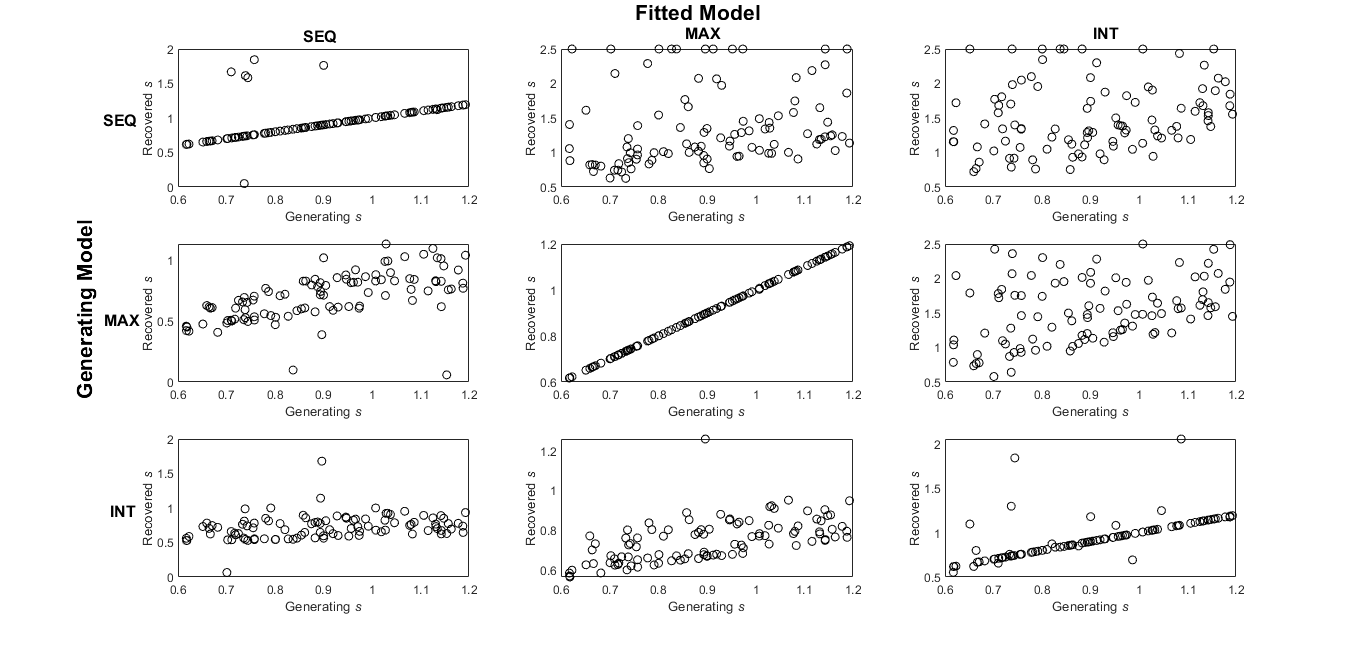


*Figure 3*. Scatter plots for *s* cross fit


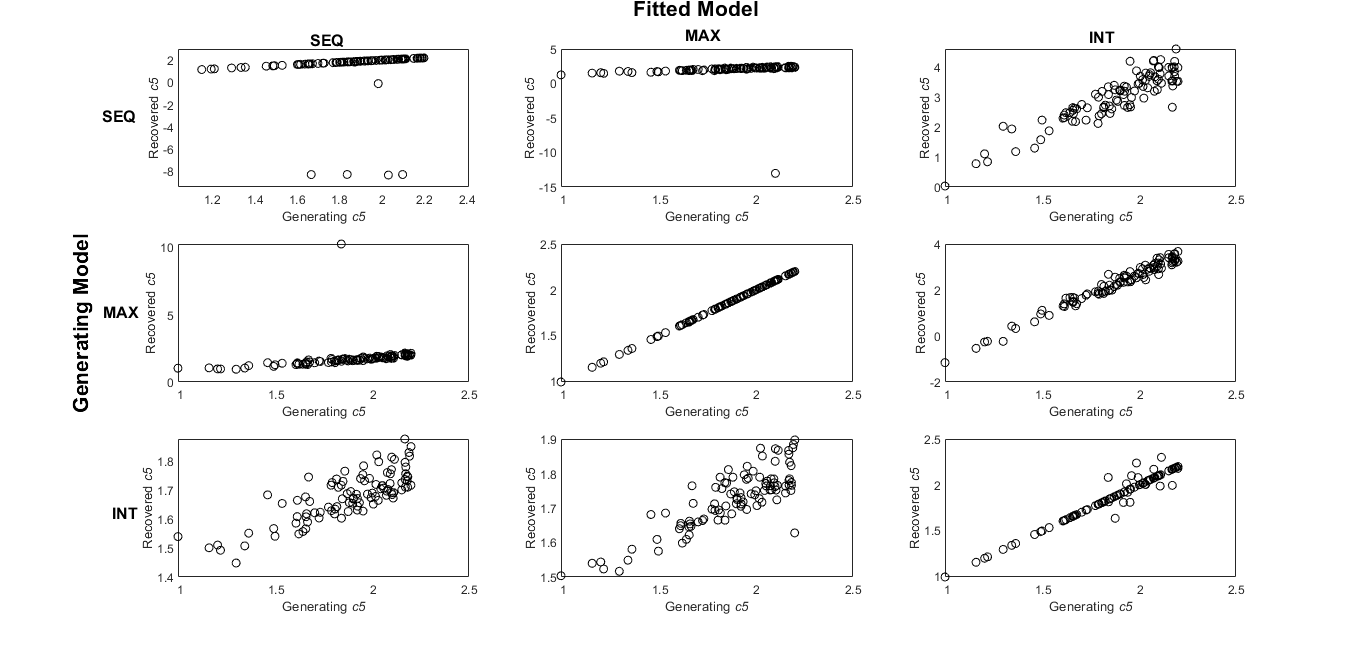


*Figure 4.* Scatter plots for *c5* cross fit


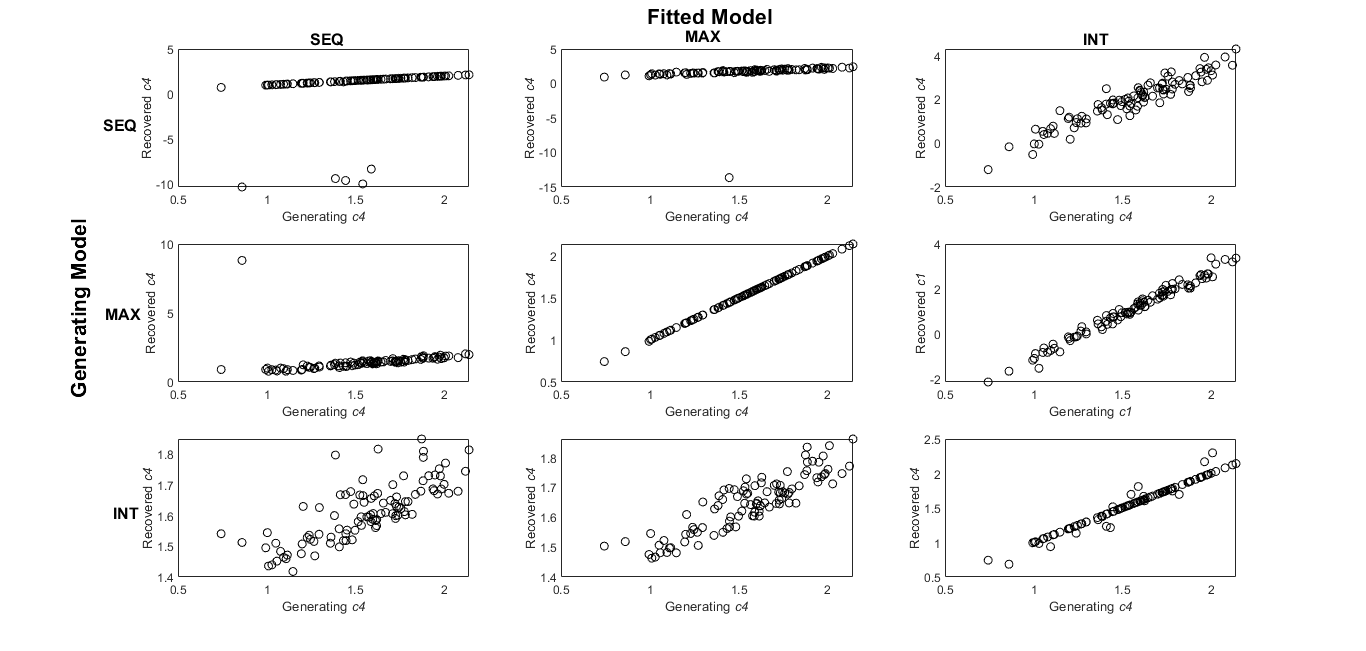


*Figure 5.* Scatterplots for *c4* cross fit

*
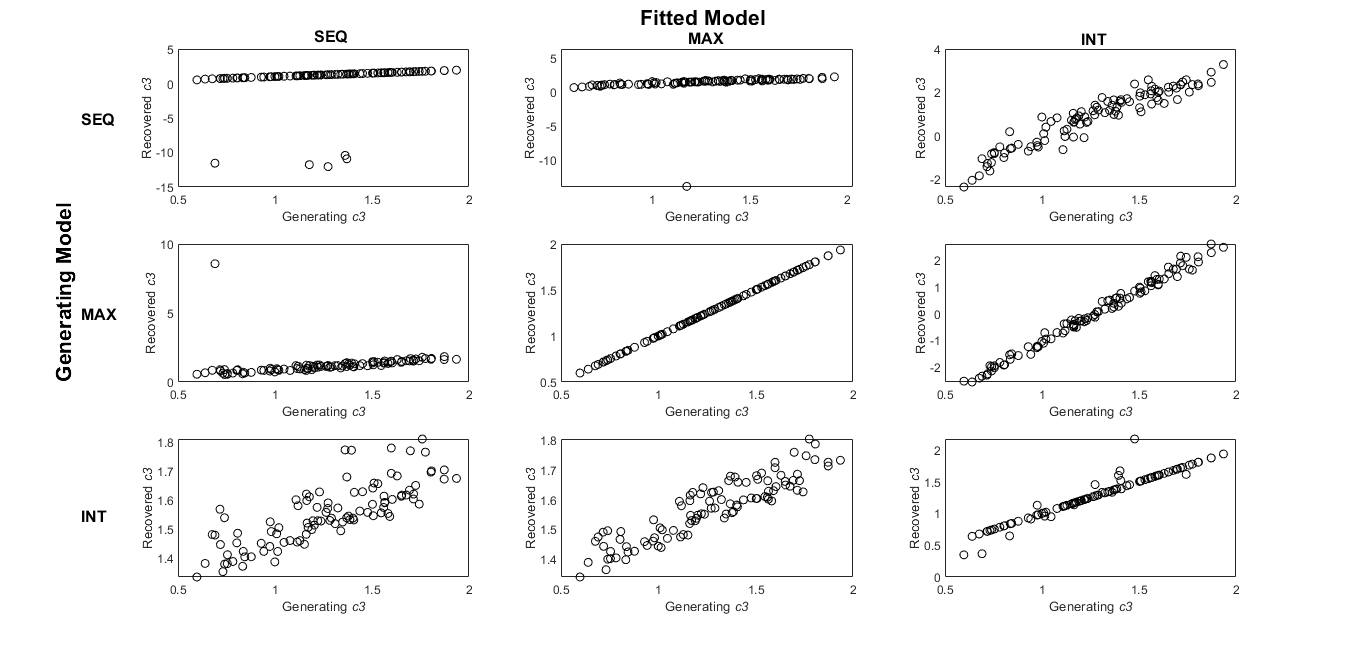
Figure 6.* Scatterplots for *c3* cross fit


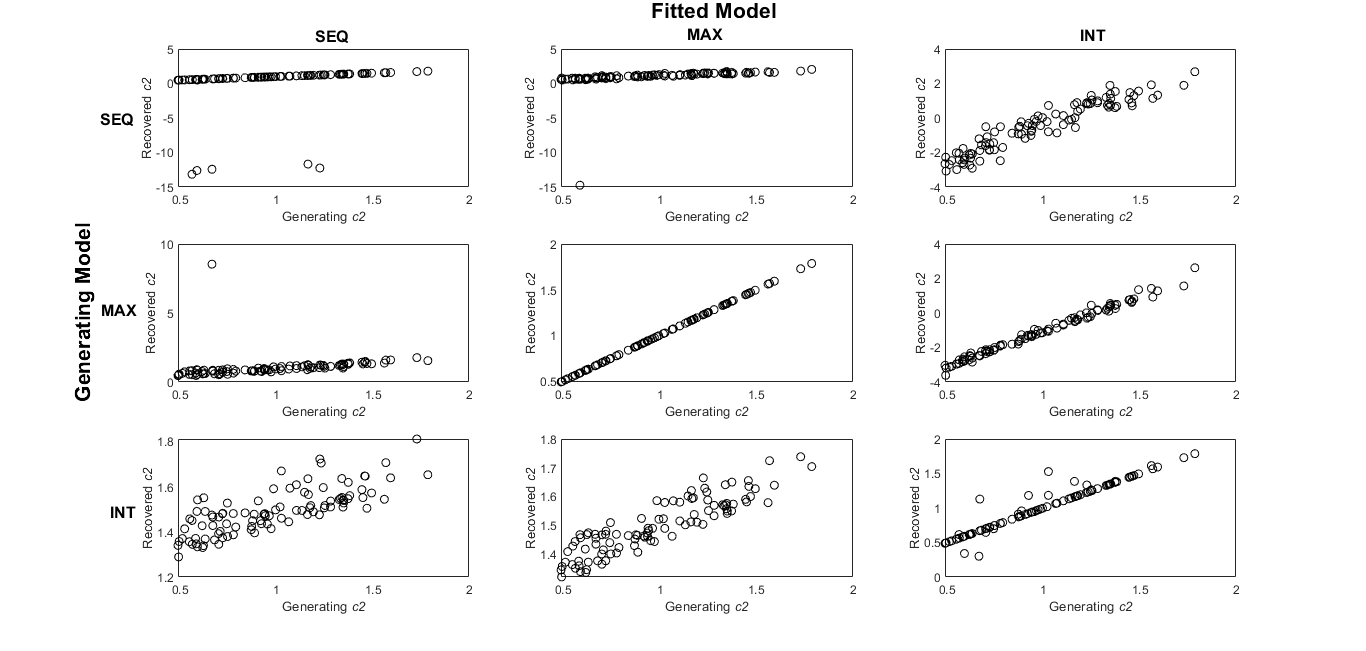


*Figure 7.* Scatterplots for *c2* cross fit


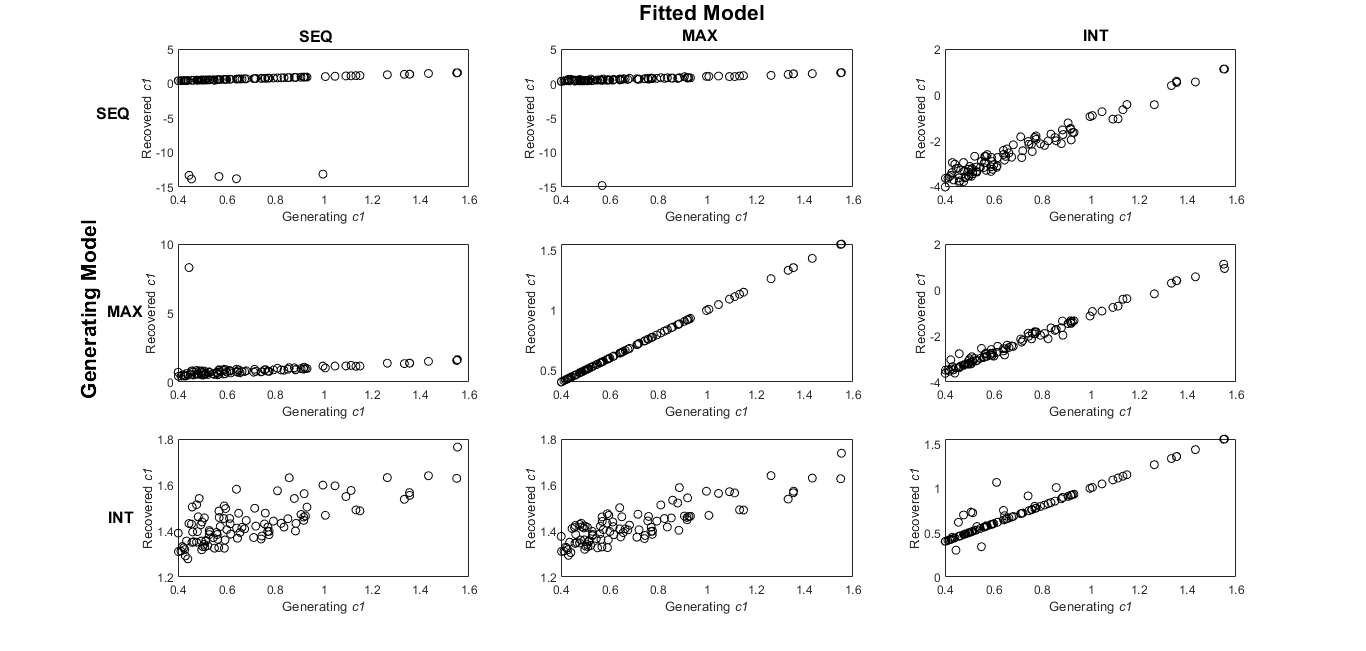


*Figure 8.* Scatterplots for *c1* cross fit

# References

Palmer, M. A., & Brewer, N. (2012). Sequential lineup presentation promotes less-biased criterion setting but does not improve discriminability. *Law and Human Behavior, 36*(3), 247-255.

Wilson, B. M., Donnelly, K., Christenfeld, N., & Wixted, J. T. (2019). Making sense of sequential lineups: An experimental and theoretical analysis of position effects. *Journal of Memory and Language, 104*, 108-125.

Wixted, J. T., Vul, E., Mickes, L., & Wilson, B. M. (2018). Models of lineup memory. *Cognitive Psychology, 105*, 81-114.
