# Supplementary material for: Do sequential lineups impair underlying discriminability?
Source: Cogn Res Princ Implic. 2020 Aug 4;5:35. doi: 10.1186/s41235-020-00234-5 (PMC7403381; doi:10.1186/s41235-020-00234-5)
Supplement: Supplementary file 3 — Additional file 3: Table S1.dt, ds, c and C for each dataset in the Palmer and Brewer (2012) corpus, estimated by the relevant models for each presentation format. Table S2. Fit statistics and dt, ds and c for each dataset in the post-2011 corpus, estimated by SDT-MAX and SDT-SEQ for simultaneous and sequential presentation respectively. [file 41235_2020_234_MOESM3_ESM.docx]

# Additional file 3

## Table S1

*d_t_, d_s_, c* and *C* for each dataset in the Palmer and Brewer (2012) corpus, estimated by the relevant models for each presentation format

|  | *d_t_* | | | | *d_s_* | | | | *c* | | | | *C* | | | |
| --- | --- | --- | --- | --- | --- | --- | --- | --- | --- | --- | --- | --- | --- | --- | --- | --- |
|  | Simultaneous | | Sequential | | Simultaneous | | Sequential | | Simultaneous | | Sequential | | Simultaneous | | Sequential | |
|  | MAX | INT | SEQ | INT | MAX | INT | SEQ | INT | MAX | INT | SEQ | INT | MAX | INT | SEQ | INT |
| Carlson et. al (2008, Exp 1) | 2.39 | 4.27 | 2.28 | 3.90 | -.27 | 0.55 | .43 | 1.89 | 1.73 | 1.86 | 2.08 | 2.54 | .53 | -0.28 | .95 | 0.59 |
| Carlson et. al (2008, Exp 2) | 1.50 | 1.80 | 1.54 | 1.99 | 1.28 | 1.88 | 1.32 | 2.70 | 1.40 | -0.22 | 1.77 | 0.95 | .65 | -1.12 | 1.00 | -0.04 |
| Clark & Davey (2005, Exp 1) | .49 | 0.80 | 1.48 | 1.50 | .90 | 1.46 | 1.13 | 1.18 | .79 | -1.30 | 1.05 | -1.18 | .55 | -1.70 | .31 | -1.93 |
| Clark & Davey (2005, Exp 2) | 1.08 | 1.40 | 1.64 | 1.70 | .80 | 1.33 | 1.18 | 1.74 | .93 | -1.10 | 1.29 | -0.74 | .39 | -1.80 | .46 | -1.59 |
| Greathouse & Kovera (2009) | .94 | 1.18 | 2.08 | 2.00 | .02 | 0.21 | -.05 | 0.02 | .75 | -1.87 | .93 | -1.75 | .28 | -2.45 | -.11 | -2.75 |
| Kneller et. al (2001) | 1.57 | 1.61 | 1.83 | 1.40 | - | - | - | - | .94 | -1.20 | 1.29 | 0.20 | .16 | -2.00 | .37 | -0.50 |
| Levi (2006) | 1.80 | 1.99 | 1.34 | 1.64 | - | - | - | - | 1.23 | -0.17 | 1.61 | 1.39 | .33 | -1.17 | .94 | 0.57 |
| Lindsay, Lea, & Fulford (1991) | 1.69 | 2.43 | 2.10 | 3.89 | .64 | 1.33 | .51 | 2.13 | 1.33 | -0.27 | 2.13 | 2.82 | .49 | -1.49 | 1.08 | 0.87 |
| Lindsay & Wells (1985) | 1.71 | 2.30 | 1.86 | 2.64 | 1.38 | 2.58 | .97 | 2.11 | 1.40 | 0.10 | 1.86 | 1.43 | .55 | -1.05 | .93 | 0.11 |
| MacLin & Phelan (2007) | 1.38 | 1.61 | 1.40 | 1.97 | - | - | - | - | 1.24 | -0.02 | 2.12 | 3.22 | .55 | -0.82 | 1.41 | 2.23 |
| MacLin et. al (2005, Exp 1) | 1.06 | 1.14 | 1.17 | 1.44 | - | - | - | - | .98 | -1.00 | 1.50 | 1.03 | .45 | -1.57 | .92 | 0.30 |
| MacLin et. al (2005, Exp 2) | 1.34 | 1.58 | 1.18 | 1.49 | - | - | - | - | 1.25 | 0.04 | 1.72 | 1.85 | .58 | -0.76 | 1.13 | 1.10 |
| Melara et. al (1989) | .34 | 0.34 | .56 | 0.70 | - | - | - | - | .47 | -3.09 | 1.62 | 1.56 | .30 | -3.26 | 1.34 | 1.21 |
| Memon & Gabbert (2003) | 1.32 | 1.46 | 1.19 | 1.70 | - | - | - | - | 1.14 | -0.37 | 2.13 | 3.34 | .48 | -1.10 | 1.54 | 2.49 |
| Parker & Ryan (1993) | 1.14 | 1.77 | .90 | 1.37 | .29 | 0.96 | -.21 | 0.25 | 1.25 | 0.20 | 1.16 | -0.01 | .68 | -0.69 | .71 | -0.69 |
| Pozzulo et. al (2008) | 1.37 | 1.56 | 1.56 | 1.90 | - | - | - | - | 1.19 | -0.19 | 1.70 | 1.62 | .51 | -0.97 | .91 | 0.67 |
| Pozzulo & Marciniak (2006) | 1.35 | 1.59 | 1.21 | 0.99 | - | - | - | - | 1.30 | 0.25 | 1.05 | -0.69 | .63 | -0.54 | .45 | -1.18 |
| Rose et. al (2005) | 1.78 | 1.94 | 1.28 | 1.39 | - | - | - | - | 1.19 | -0.32 | 1.36 | 0.43 | .30 | -1.29 | .72 | -0.27 |
| Sporer (1993) | 1.11 | 1.25 | 1.34 | 1.64 | - | - | - | - | .97 | -1.03 | 1.50 | 0.99 | .41 | -1.66 | .83 | 0.17 |
| Steblay et. al (2011) | .30 | 0.32 | .19 | 0.22 | - | - | - | - | 1.53 | 1.17 | 1.54 | 1.21 | 1.38 | 1.01 | 1.45 | 1.10 |
| Wells & Pozzulo (2006) | .74 | 0.85 | .19 | 0.16 | - | - | - | - | 1.26 | 0.18 | 1.22 | -0.05 | .89 | -0.25 | 1.12 | -0.13 |
| Wilcock et. al (2005) | 1.07 | 1.32 | 1.78 | 2.05 | - | - | - | - | 1.19 | -0.09 | 1.61 | 1.16 | .66 | -0.75 | .72 | 0.14 |

## Table S2

Fit statistics and *d_t_, d_s_* and *c* for each dataset in the post-2011 corpus, estimated by SDT-MAX and SDT-SEQ for simultaneous and sequential presentation respectively

|  |  | *χ*^2^(1) | | *p* | | *d_t_* | | *d_s_* | | *c* | |
| --- | --- | --- | --- | --- | --- | --- | --- | --- | --- | --- | --- |
| Study | Condition | sim | seq | sim | seq | sim | seq | sim | seq | sim | seq |
| Pica & Pozzulo (2017) | - | .18 | .90 | .68 | .34 | 2.37 | 2.22 | - | - | 1.31 | 1.80 |
| Flowe et al. (2016) | Upright | 2.60 | 1.14 | .11 | .29 | 1.64 | 1.38 | - | - | 1.33 | 1.45 |
|  | Inverted | .56 | 1.03 | .46 | .31 | .77 | .58 | - | - | 1.00 | 1.10 |
| Carlson et al. (2016) | Backloading | 10.29 | .35 | <.01 | .56 | 1.05 | 1.32 | - | - | 1.16 | 1.28 |
|  | No backloading | .26 | .92 | .61 | .17 | 1.07 | 1.17 | - | - | .83 | 1.09 |
| Pozzulo et al. (2016) | - | 3.23 | .94 | .07 | .33 | 1.95 | 1.28 | - | - | 1.28 | 1.47 |
| Sucic et al. (2015)^a^ | - | 2.45 | 8.67 | .12 | <.01 | 1.24 | 1.43 | 1.09 | 1.16 | 1.02 | 1.39 |
| Carlson & Carlson (2014) | No weapon, no feature | .84 | .18 | .36 | .68 | .89 | .77 | -.67 | -.48 | .82 | .89 |
|  | No weapon, feature | .02 | .01 | .90 | .91 | .48 | .20 | -.15 | -.49 | .89 | .73 |
|  | Weapon, no feature | 1.11 | 3.19 | .29 | .07 | 1.21 | .52 | -.19 | -.11 | 1.03 | .83 |
|  | Weapon, feature | 1.76 | 10.09 | .18 | <.01 | .56 | .76 | -.11 | -.51 | .97 | .80 |
| Pozzulo et al. (2013) | - | .18 | 4.46 | .67 | .03 | 1.78 | 1.30 | - | - | 1.15 | 1.48 |
| Mickes et al. (2012) | Experiment 1a | .23 | .10 | .63 | .75 | 2.02 | 1.29 | - | - | 1.51 | 1.60 |

^a^ Sucic et al. (2015) included an additional “don’t know” response option. We excluded “don’t know” responses when analysing the data, adjusting the total number of target present and target absent trials accordingly.

# References

Carlson, C. A., & Carlson, M. A. (2014). An evaluation of lineup presentation, weapon presence, and a distinctive feature using ROC analysis. *Journal of Applied Research in Memory and Cognition, 3*(2), 45-53.

Carlson, C. A., Carlson, M. A., Weatherford, D. R., Tucker, A., & Bednarz, J. (2016). The effect of backloading instructions on eyewitness identification from simultaneous and sequential lineups. *Applied Cognitive Psychology, 30*(6), 1005-1013.

Carlson, C. A., Gronlund, S. D., & Clark, S. E. (2008). Lineup Composition, Suspect Position, and the Sequential Lineup Advantage. *Journal of Experimental Psychology: Applied, 14*(2), 118-128.

Clark, S. E., & Davey, S. L. (2005). The Target-to-Foils Shift in Simultaneous and Sequential Lineups. *Law and Human Behavior, 29*(2), 151-172.

Flowe, H. D., Smith, H. M., Karoglu, N., Onwuegbusi, T. O., & Rai, L. (2016). Configural and component processing in simultaneous and sequential lineup procedures. *Memory, 24*(3), 306-314.

Greathouse, S. M., & Kovera, M. B. (2009). Instruction bias and lineup presentation moderate the effects of administrator knowledge on eyewitness identification. *Law and Human Behavior, 33*(1), 70-82.

Kneller, W., Memon, A., & Stevenage, S. (2001). Simultaneous and sequential lineups: Decision processes of accurate and inaccurate eyewitnesses. *Applied Cognitive Psychology, 15*(6), 659-671.

Levi, A. M. (2006). An analysis of multiple choices in MSL lineups, and a comparison with simultaneous and sequential ones. *Psychology, Crime & Law, 12*(3), 273-285.

Lindsay, R., & Wells, G. L. (1985). Improving eyewitness identifications from lineups: Simultaneous versus sequential lineup presentation. *Journal of Applied Psychology, 70*(3), 556-564.

Lindsay, R. C., Lea, J. A., & Fulford, J. A. (1991). Sequential lineup presentation: Technique matters. *Journal of Applied Psychology, 76*(5), 741-745.

MacLin, O. H., & Phelan, C. M. (2007). PC_Eyewitness: Evaluating the New Jersey method. *Behavior Research Methods, 39*(2), 242-247.

Maclin, O. H., Zimmerman, L. A., & Malpass, R. S. (2005). PC_Eyewitness and the Sequential Superiority Effect: Computer-Based Lineup Administration. *Law and Human Behavior, 29*(3), 303-321.

Melara, R. D., & De Witt-Rickards, T. S. (1989). Enhancing Lineup Identification Accuracy: Two Codes Are Better Than One. *Journal of Applied Psychology, 74*(5), 706.

Memon, A., & Gabbert, F. (2003). Unravelling the effects of sequential presentation in culprit-present lineups. *Applied Cognitive Psychology, 17*(6), 703-714.

Mickes, L., Flowe, H. D., & Wixted, J. T. (2012). Receiver operating characteristic analysis of eyewitness memory: Comparing the diagnostic accuracy of simultaneous versus sequential lineups. *Journal of Experimental Psychology: Applied, 18*(4), 361-376.

An attempt to reduce guessing behavior in children's and adults' eyewitness identifications, 17 C.F.R. (1993).

Pica, E., & Pozzulo, J. (2017). The elimination-plus lineup: Testing a modified lineup procedure with confidence. *Journal of Investigative Psychology and Offender Profiling, 14*(3), 294-306.

Pozzulo, J. D., Dempsey, J., Corey, S., Girardi, A., Lawandi, A., & Aston, C. (2008). Can a lineup procedure designed for child witnesses work for adults? Comparing simultaneous, sequential, and elimination lineup procedures. *Journal of Applied Social Psychology, 38*(9), 2195-2209.

Pozzulo, J. D., Dempsey, J., & Pettalia, J. (2013). The Z Generation: Examining Perpetrator Descriptions and Lineup Identification Procedures. *Journal of Police and Criminal Psychology, 28*(1), 63-74.

Pozzulo, J. D., Reed, J., Pettalia, J., & Dempsey, J. (2016). Simultaneous, sequential, elimination, and wildcard: A comparison of lineup procedures. *Journal of Police and Criminal Psychology, 31*(1), 71-80.

Rose, R. A., Bull, R., & Vrij, A. (2005). Non-biased lineup instructions do matter--A problem for older witnesses. *Psychology, Crime & Law, 11*(2), 147-159.

Sporer, S. L. (1993). Eyewitness identification accuracy, confidence, and decision times in simultaneous and sequential lineups. *Journal of Applied Psychology, 78*(1), 22-33.

Steblay, N. K., Dietrich, H. L., Ryan, S. L., Raczynski, J. L., & James, K. A. (2011). Sequential lineup laps and eyewitness accuracy. *Law and Human Behavior, 35*(4), 262-274.

Sučić, I., Tokić, D., & Ivešić, M. (2015). Field study of response accuracy and decision confidence with regard to lineup composition and lineup presentation. *Psychology, Crime and Law, 21*(8), 798-819.

Wells, E. C., & Pozzulo, J. D. (2006). Accuracy of eyewitnesses with a two-culprit crime: Testing a new identification procedure. *Psychology, Crime & Law, 12*(4), 417-427.

Wilcock, R. A., Bull, R., & Vrij, A. (2005). Aiding the Performance of Older Eyewitnesses: Enhanced Non-Biased Line-Up Instructions and Line-Up Presentation. *Psychiatry, Psychology and Law, 12*(1), 129-140.
