# Supplementary material for: Do sequential lineups impair underlying discriminability?
Source: Cogn Res Princ Implic. 2020 Aug 4;5:35. doi: 10.1186/s41235-020-00234-5 (PMC7403381; doi:10.1186/s41235-020-00234-5)
Supplement: Supplementary file 4 — Additional file 4: Supplement 1. Fitting SDT-MAX to Simultaneous Lineup Data. [file 41235_2020_234_MOESM4_ESM.zip › simseq_Supplement_SDT-MAX_Walkthrough_ESM.docx]

Supplement 1

# Fitting SDT-MAX to Simultaneous Lineup Data

First, download the R script “Supplement_SDT-MAX.R”, which can be found at https://osf.io/jw64c/. This file contains functions for fitting an unequal variance version of SDT-MAX, also known as the Independent Observations model, to simultaneous lineup data. This document will explain what each piece of the code does and how it works together.

## Data Structure

A critical aspect of working with this code is formatting observed data in a way that is appropriate for the likelihood functions that predict data according to the model. The data from this study (Kaesler, Dunn, Ransom & Semmler, in preparation) is formatted below:

# Confidence Bin 100-91 90-81 80-66 65-51 50-0 Reject
# Criterion c5 c4 c3 c2 c1
# Target IDs (TID) 24 25 30 9 11 NaN
# TP Foil IDs + TID (TD) 24 26 35 13 22 19
# Target Absent Foil IDs (FA) 4 11 25 16 24 61

The criteria are organised from most conservative ($c_{5}$) to least conservative ($c_{1}$); $c_{1}$ is the choose/no-choose threshold. The last column contains all rejections. There are no rejections associated with target identification decisions, so NaN appears in that cell. Incorrect target present (TP) rejections appear in the last cell of the TD row, which contains all detections on TP trials. Correct rejections appear in the last cell of the FA row, which contains all detections on TA trials. Summing the TD row should give the total number of TP trials, summing the TA row should give the total number of TA trials.

The code below sets up the observed data as a matrix and also specifies the lineup size, which is used by the likelihood functions.

obsData <- matrix(data = c(24,25,30,9,11,NaN,
 24,26,35,13,22,19,
 4,11,25,16,24,61),

 nrow = 3,
 ncol = 6,
 byrow = TRUE)

n <- 6 #lineup size

## Likelihood Functions

The next section of code contains the three functions that predict each outcome in our data, TID, TD and FA, according to the MAX decision rule. These functions take the model parameters and lineup size as input, and return probabilities as output. Derivations for the relevant equations can be found in Appendix A.

# Probability of Target ID (TID) on TP trials according to MAX model
QT <- function(c,d,s,n){
 m <- function(x) dnorm(x,mean = d, sd = s)*pnorm(x)^(n-1)
 p <- rep(0,length(c))
 for (i in 1:length(c)){
 a <- integrate(m,c[i],15)
 p[i] <- a$value
 }
 return(p)
}

# Probability of detection (TD) on TP trials MAX model
# This is any detection, both TID and TP foil ID
TP <- function(c,d,s,n){
 p <- rep(0,length(c))
 for (i in 1:length(c)){
 p[i] <- pnorm(((c[i])-d)/s)*pnorm(c[i])^(n-1)
 }
 p <- 1 - p
 return(p)
}

# Probability of TA foil ID (FA) MAX model
TA <- function(c,n){
 p <- rep(0,length(c))
 for (i in 1:length(c)){
 p[i] = pnorm(c[i])^n
 }
 p = 1 - p
 return(p)
}

These functions are called by a wrapper function genPred. It takes a vector of parameters, the observed data and the lineup size as input and returns predicted data as output in the same format as the observed data. The likelihood functions return probabilities, so genPred converts these to counts by multiplying by the total number of observed TP/TA lineups. This is only reason for passing the observed data to genPred.

genPred <- function(pars, obsData, n){

 # Unpack vector of parameters for use by likelihood functions
 c <- pars[1:(length(pars)-2)]
 d <- pars[length(pars)-1] # second-to-last parameter is always d
 s <- tail(pars,1) # last parameter is always s

 # Calculate total number of TP and TA lineups
 totalTP <- sum(obsData[2,])
 totalTA <- sum(obsData[3,])

 TID <- QT(c(c, -15),d,s,n)
 TID <- c(TID[1],diff(TID))

 TD <- c(TP(c,d,s,n),1)
 TD <- c(TD[1],diff(TD))

 FA <- c(TA(c,n),1)
 FA <- c(FA[1],diff(FA))

 # Convert proportions to counts
 TID <- TID*totalTP
 TD <- TD*totalTP
 FA <- FA*totalTA

 predData <- rbind(TID,TD,FA)
 rownames(predData) <- c()

 return(predData)
}

## Chi-Square Goodness-of-Fit Test

We use a $\chi^{2}$ test to assess model fit. The chiSq function takes a vector of parameters, the observed data and the lineup size as input. It passes these to genPred to generate predicted data, which is compared to the observed data, returning a $\chi^{2}$ value as output.

chiSq <- function(pars,obsData,n){

 predData <- genPred(pars,obsData,n)
 lastcell <- ncol(obsData)
 ncrit <- ncol(obsData)-1 #number of criteria
 f <- rep(0,nrow(obsData)+2) #for storing and summing chi-sq fit value

 for (i in 1:ncrit){

 # TID
 a <- predData[1,i]
 b <- obsData[1,i]
 f[1] <- f[1] + (b-a)^2/a

 # Foil ID on TP lineup
 a <- predData[2,i]-predData[1,i]
 b <- obsData[2,i]-obsData[1,i]
 f[2] = f[2] + (b-a)^2/a

 # FA
 a <- predData[3,i]
 b <- obsData[3,i]
 f[3] <- f[3] + (b-a)^2/a
 }

 # Incorrect Rejection TP
 a <- predData[2,lastcell]
 b <- obsData[2,lastcell]
 f[4] <- (b-a)^2/a

 # Correct Rejection TA
 a <- predData[3,lastcell]
 b <- obsData[3,lastcell]
 f[5] <- (b-a)^2/a

 f <- sum(f)

 return(f)
}

We have now defined the functions necessary for fitting SDT-MAX to the data; QT, TP and TA are the likelihood functions that give the probability of TID, TD and FA respectively according to the MAX model, genPred is a wrapper function that calls these functions and returns predicted data in the same format as the observed data, and chiSq is the function that calls genPred and returns a goodness-of-fit value that reflects how close the predicted data is to the observed data.

## Setting Starting Values for the Optimisation

We now turn to some setup for the optimisation step. One aspect of model fitting that can be difficult is setting appropriate starting values. If a parameter space is particular “lumpy”, the best fitting parameter values recovered by optimisation may change depending on which starting values are used. This is because the optimisation routine may fall in to local minima near the starting values when searching the parameter space rather than finding the global minimum. Additionally, arbitrarily chosen starting values may sometimes lie outside the feasible region of the function being optimised, in which case the optimisation routine will not run.

We have attempted to mitigate these issues by setting random-like starting values for the criteria and plausible starting values for $d_{t}$ and $s_{t}$, i.e. values that are close to those typically recovered in recognition memory experimenets. Note that this method for setting starting values may not work for every possible dataset, particularly those where participants behaved unusually. The function startVals takes the observed data and lineup size as input and returns a vector of starting values as output. The order of the parameters in this vector is important, as genpred unpacks this vector in the order $c_{max},...,c_{1},d_{t},s_{t}$ for passing to the likelihood functions.

startVals <- function(obsData,n) {

 ncrit <- ncol(obsData)-1 #number of criteria

 # Random-like criteria starting points
 c0 <- obsData[nrow(obsData),]
 c0[length(c0)] <- max(c(c0,0.5))
 a0 <- cumsum(c0)/sum(c0)
 c0 <- qnorm((1-a0)^(1/n))
 c0 <- c0[1:length(c0)-1]

 # Concatenate to form starting parameter vector in order accepted by genpred
 x0 = c(c0,1.5,1) #cMax, ..., c1, dt, s

 return(x0)
}

# Call function to generate starting values
x0 <- startVals(obsData,n)
x0

## [1] 2.590990 2.084184 1.606439 1.399098 1.124807 1.500000 1.000000

## Inequality Constraints

We now set up the inequality constraints to ensure that the criteria do not “cross over” during optimsation. For example, unconstrained optimisation might converge on a solution where $c_{4}$ is greater than $c_{5}$, which is not interpretable.

In our case, we have a vector of parameters ordered ($c_{5},c_{4},c_{3},c_{2},c_{1},d_{t},s_{t}$). We can consider this a vector labeled ($x_{1},...,x_{7}$). We want to satisfy the inequality constraint that $x_{1}\geq x_{2}\geq x_{3}\geq x_{4}\geq x_{5}$. We have no inequality constrains on $x_{6}$ ($d_{t}$) or $x_{7}$ ($s$). Expressed as a series of linear equations, our inequality constraints are:

$$x_{1}-x_{2}\geq0;x_{2}-x_{3}\geq0;x_{3}-x_{4}\geq0;x_{4}-x_{5}\geq0;$$

The constrOptim function we use for the optimisation takes inequality constraints in the form $Ax\geq b$, where $Ax$ is a $k*p$ matrix of $k$ inequality constrains and $p$ parameters, and $b$ is a vector of the constraints to be satisified. We have four inequality constrains ($k=4$) and seven parameters ($p=7$), so $Ax$ is a 4 x 7 matrix, and $b$ is a vector of length four.

To represent our linear equations in matrix form, conforming to the requirement that $Ax\geq b$, we need to generate the following matrix for $Ax$:

## [,1] [,2] [,3] [,4] [,5] [,6] [,7]
## [1,] 1 -1 0 0 0 0 0
## [2,] 0 1 -1 0 0 0 0
## [3,] 0 0 1 -1 0 0 0
## [4,] 0 0 0 1 -1 0 0

and the following vector for $b$:

## [1] 0 0 0 0

This can achieved for any number of criteria with the following code:

# Set inequality constraint matrix for criteria, stops them crossing over
ncrit <- ncol(obsData)-1 # number of criteria
nparam <- length(x0) # number of parameters
A <- matrix(0,ncrit-1,nparam); # k x p matrix
b <- rep(0,ncrit-1) # vector of >= constraints to satisfy
for (i in 1:ncrit-1) {
 A[i,i] <- 1; A[i,i+1] <- -1
}

## Optimisation

We now use the R function constrOptim to minimise the function chiSq. constrOptim takes the vector of starting values theta = x0 and passes it as the first argument (pars) of the function to be minimised, f = chiSq. It takes the constraint matrix ui = A and the constraint vector ci = b. The last two input arguments to the function are the extra inputs required by chiSq; obsData and the lineup size n. constrOptim returns a list including the value of chiSq at the optimal solution and the best fitting parameters.

Consult the optim documentation for information about the remaining input arguments, which are options for the optimisation routine.

# Minimise chiSq function using the starting values and constraints defined above
out <- constrOptim(theta = x0, f = chiSq, grad = NULL, ui = A, ci = b, mu = 1e-04, method = "Nelder-Mead", outer.iterations = 100, obsData = obsData, n = n)

When we run the optimisation it starts with the parameters in x0 and, at each iteration, it searches for a new set of parameter values that minimise $\chi^{2}$. It usually stops when it converges on an optimal solution, but it may stop for other reasons without converging, such as reaching the maximum number of iterations specified in the options.

## Output

Once the optimisation has finished we can examine the list stored in out. out$par contains the best fitting parameter values in the same order as specified in x0. out$value is the smallest $\chi^{2}$ value at the optimal solution, associated with the parameters in out$par. out$counts shows how many times chiSq was called, i.e. how many iterations were required to find the optimal solution. out$convergence shows whether the optimisation converged. Zero indicates that it did converge in this case. Consult the documentation for optim for information on other convergence messages that may indicate unsuccessful convergence.

out

## $par
## [1] 2.7214683 2.2022708 1.6910396 1.4885855 1.1593367 1.8250110 0.9384431
##
## $value
## [1] 13.43876
##
## $counts
## function gradient
## 1004 NA
##
## $convergence
## [1] 0
##
## $message
## NULL
##
## $outer.iterations
## [1] 3
##
## $barrier.value
## [1] 0.0002934577

Our degrees of freedom for calculating a *p*-value for our $\chi^{2}$ test are based on the format of our data and our number of parameters. The degrees of freedom are calculated as the number rows in the dataset multiplied by the number of criteria, minus the number of parameters estimated. At least one degree of freedom is required to fit the model; the output of a saturated model with zero degrees of freedom is not interpretable. The code below uses pchisq, the $\chi^{2}$ distribution function, to find *p* for our minimum $\chi^{2}$ value with the appropriate degrees of freedom. The *p*-value is non-significant, so we can conclude that the model fit the data.

# Degrees of freedom and p-value
df <- (nrow(obsData)*ncrit) - nparam
pval <- pchisq(out$value, df = df, lower.tail = FALSE)
pval

## [1] 0.09761866

We can pass the best-fitting parameters in out$par to genpred to get the predicted data associated with the optimal solution. This can be used for plotting model ROCs. Additionally, if the model did not fit, it can be useful to compare the observed and predicted data to see where it failed.

Finally, we can extract the parameters from out$par and collate all information relevant to our results in to a list to make it easy to read at a glance.

# Predicted Data - Useful for plotting model ROCs
predData <- genPred(out$par, obsData, n)
predData[1,ncol(predData)] <- NaN #replace meaningless number in reject cell of pred Data with NaN

# Collate in to list for easy viewing
results <- list(
 c = out$par[1:(length(out$par)-2)], # same as extracting pars in genpred
 d = out$par[length(out$par)-1],
 s = tail(out$par,1),
 x0 = x0,
 n = n,
 nparam = nparam,
 fitVal = out$value,
 df = df,
 pval = pval,
 obs = obsData,
 pred = predData,
 converge = out$convergence)

results

## $c
## [1] 2.721468 2.202271 1.691040 1.488586 1.159337
##
## $d
## [1] 1.825011
##
## $s
## [1] 0.9384431
##
## $x0
## [1] 2.590990 2.084184 1.606439 1.399098 1.124807 1.500000 1.000000
##
## $n
## [1] 6
##
## $nparam
## [1] 7
##
## $fitVal
## [1] 13.43876
##
## $df
## [1] 8
##
## $pval
## [1] 0.09761866
##
## $obs
## [,1] [,2] [,3] [,4] [,5] [,6]
## [1,] 24 25 30 9 11 NaN
## [2,] 24 26 35 13 22 19
## [3,] 4 11 25 16 24 61
##
## $pred
## [,1] [,2] [,3] [,4] [,5] [,6]
## [1,] 23.45591 23.279170 25.80194 8.672849 10.35475 NaN
## [2,] 25.45453 28.471077 36.23977 13.703964 17.90724 17.22342
## [3,] 2.72695 8.570629 23.01633 14.454322 28.14878 64.08299
##
## $converge
## [1] 0
